# Bayesian inference of epidemiological parameters from transmission experiments

**DOI:** 10.1038/s41598-017-17174-8

**Published:** 2017-12-01

**Authors:** Ben Hu, Jose L. Gonzales, Simon Gubbins

**Affiliations:** 1 The Pirbright Institute, Ash Road, Pirbright, Surrey, GU24 0NF UK; 2 Centre for Complexity Science, University of Warwick, Coventry, CV4 7AL UK; 3 Wageningen BioVeterinary Research, Houtribweg 39, 8221 RA Lelystad, The Netherlands

## Abstract

Epidemiological parameters for livestock diseases are often inferred from transmission experiments. However, there are several limitations inherent to the design of such experiments that limits the precision of parameter estimates. In particular, infection times and latent periods cannot be directly observed and infectious periods may also be censored. We present a Bayesian framework accounting for these features directly and employ Markov chain Monte Carlo techniques to provide robust inferences and quantify the uncertainty in our estimates. We describe the transmission dynamics using a susceptible-exposed-infectious-removed compartmental model, with gamma-distributed transition times. We then fit the model to published data from transmission experiments for foot-and-mouth disease virus (FMDV) and African swine fever virus (ASFV). Where the previous analyses of these data made various assumptions on the unobserved processes in order to draw inferences, our Bayesian approach includes the unobserved infection times and latent periods and quantifies them along with all other model parameters. Drawing inferences about infection times helps identify who infected whom and can also provide insights into transmission mechanisms. Furthermore, we are able to use our models to measure the difference between the latent periods of inoculated and contact-challenged animals and to quantify the effect vaccination has on transmission.

## Introduction

Transmission experiments offer a wealth of data from which we can estimate important epidemiological parameters for livestock diseases. The basic reproduction number (*R*_0_), defined as the average number of secondary cases caused by an infected individual in a totally susceptible population^1^, transmission rates and latent and infectious period durations can all be inferred from such experiments. This is particularly important for high consequence animal diseases, such as avian influenza, foot-and-mouth disease, African swine fever or classical swine fever. For these diseases, collecting data during outbreaks is often hampered by a lack of capacity in parts of the world where they are endemic and by a conflict between the requirements for expeditious disease control versus information gathering when epidemics occur in disease-free countries. Consequently, transmission experiments are often the primary source of data from which to infer transmission, latent period or infectious period parameters. Estimating these parameters directly from the data, rather than making simplifying assumptions or relying on expert opinion^2^, lends good strength to any conclusions drawn.

Many transmission experiments follow a similar design in which the pathogen of interest is introduced to a group of animals, usually by some inoculated seed animals, and the subsequent spread through the rest of the population is recorded^3^. Generally, all individuals are monitored for clinical signs and biological samples are collected at regular intervals to detect the pathogen (i.e. to determine whether or not transmission has occurred). However, this design means that the latent periods for the inoculated animals are only known in the range between the last negative and first positive samples. More importantly, the infection times and latent periods of the in-contact animals are unobserved. Finally, the infectious periods are similarly unobserved and may also be right-censored due to the pre-determined experiment duration^4^ or welfare grounds, in the case of severe disease^5^. In addition, the test used to detect the pathogen is assumed to be perfect, so that the test results provide an accurate picture of if and when an animal is infected. The numbers of animals that can be used in this type of experiment are also limited on logistical (numbers of animals that can be housed), cost (experiments in high containment are expensive) and ethical (with the aim of reducing the number of animals used in experiments) grounds. Even though the number of animals used are limited, the results obtained via transmission experiments are nonetheless a reasonable approximation to field observations^6^. Furthermore, scaling experimental results to the herd level is facilitated by the assumption of frequency-dependent transmission used when analysing the experimental data, which has been shown to be appropriate for farm animals^7^.

Many existing methods for analysing such data do not account for these issues directly, instead making assumptions to overcome them. The final size (FS) method^3^ has been used to estimate *R*_0_ for many diseases, such as Aujeszky’s disease^8^, avian influenza^9^ and foot-and-mouth-disease^10,11^, but offers no inferences on other epidemiological parameters. This uses a compartmental model and, as the name implies, only considers the numbers of animals in each compartment at the end of the experiment, discarding any information that can be gained from the progress of the epidemic. This method assumes that the final size has been reached by the end of the experiment, but this is not always applicable^4^. Furthermore, if all animals become infected, this method returns *R*_0_ = ∞ (see, for example, refs^9,11^). In this case, it can still be useful for qualitative comparisons between different treatment groups.

Another widely used method, first described in a study on classical swine fever virus^12^, makes more use of the data by considering the state of the transmission chain in discrete time steps, where numbers of susceptible and infectious animals are known. Transmission rates can be inferred using a generalised linear model (GLM). Infectious periods can also be inferred using survival analysis if they are not all right-censored, but latent periods, if included, must be assumed to be fixed and known.

Bayesian methods^13–15^ provide an ideal framework in which to overcome the problems in this experimental design, without needing to make the assumptions required to implement the FS or GLM methods. Incorporating the unobserved infection times and latent periods as nuisance parameters in the model allows us to draw inferences about them. This is potentially useful in terms of identifying who infected whom during an experiment. It can also help understand transmission mechanisms, for example, by comparing viral shedding patterns with times of infection. The use of informative priors allows us to estimate infectious periods even for censored data and also helps restrict the transmission rate to biologically reasonable parameter space. Here we apply a Bayesian framework to analyse transmission experiments using a stochastic susceptible-exposed-infectious-recovered (SEIR) type model, inferring latent and infectious period distributions and the transmission parameter simultaneously.

We first present a simple generic form of the model and the inference methodology. We then fit the models to experiments on the transmission of foot-and-mouth disease virus (FMDV)^4,11^ and of African swine fever virus (ASFV)^5,16^, two contagious pathogens of high socio-economic importance. Foot-and-mouth disease has caused major disruptive epidemics in 2001 in the UK^17^ and in 2010 in the Republic of Korea^18^ and Japan^19^. African swine fever is another major threat, currently circulating in eastern Europe with a high risk of becoming endemic and of onward transmission into unaffected areas^20^. We discuss the differences in the experimental designs, show how the model can be adapted to account for these features and compare the inferences against those obtained by the original analyses using the FS and GLM methods. We also use simulated data to explore the framework and to assess the robustness of the inferences about the unobserved processes. We use the models to see if the route of infection, whether by inoculation or contact, has an effect on the latent period of the animal. By fitting the models to data on FMDV in both vaccinated and unvaccinated pigs, we can also quantify the impact that vaccination has on transmission.

## Materials and Methods

### Generic data and model

**Figure 1:**
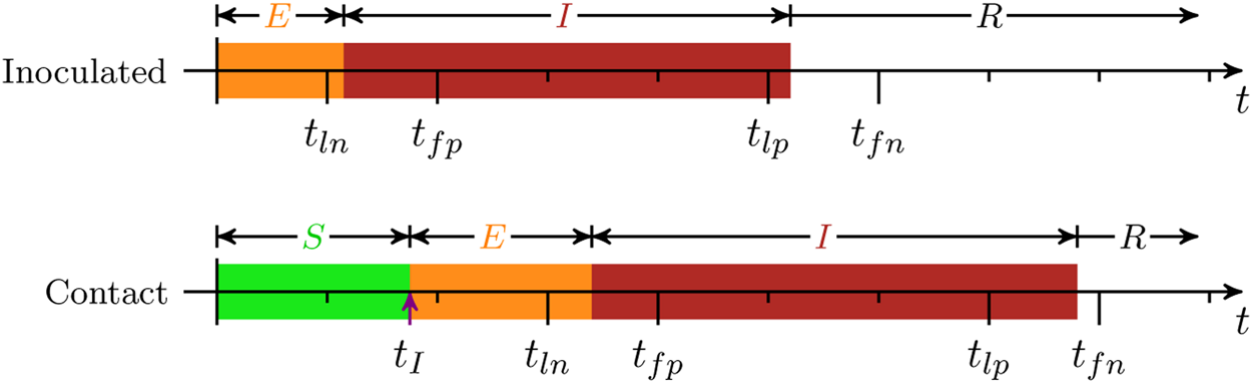
Schematic of the data and model. Animals are classified according to whether they are susceptible (*S*), exposed (i.e. infected but not yet infectious) (*E*), infectious (*I*) or recovered (*R*). The data provide the times of last negative, *t*_*ln*_, and first positive, *t*_*fp*_, samples, which provide constraints on the inferred infection time, *t*_*I*_, and latent period, *E*. Similarly, the times of last positive, *t*_*lp*_, and first negative, *t*_*fn*_, constrain the infectious period, *I*.

In general, a transmission experiment begins with some number of animals being inoculated with the pathogen of interest. These will then be allowed to mix with uninfected animals in a controlled environment. All animals are monitored for clinical signs of the disease as well as having samples, for example, blood or oropharyngeal fluid (OPF), taken at regular intervals and tested for presence of the pathogen. These samples give either a positive or negative indicator for infection at each time point. We use the times of last negative, *t*_*ln*_, first positive, *t*_*fp*_, last positive, *t*_*lp*_, and, if it exists, the first negative after recovery, *t*_*fn*_ and whether the animal was culled or not before the end of the experiment, *t*_end_, as inputs for the model (Fig. 1).

We use an SEIR model^21^, classifying animals as either (S)usceptible, (E)xposed (i.e. infected, but not yet infectious), (I)nfectious or (R)ecovered (or removed) (Fig. 1). The transition times from the exposed to infectious and infectious to recovered compartments correspond to the latent and infectious periods of the virus, respectively. These are often, though not always, assumed to be exponentially-distributed for mathematical convenience. To allow the transition probabilities to more realistically depend on the length of time already spent in the compartment we assume they follow gamma distributions^21,22^. This also has the added bonus of computationally cheap simulation. The probability density function is given by:

1 $$f(x;k,\mu )=\frac{1}{{\rm{\Gamma }}(k)}\frac{k}{\mu }{(\frac{kx}{\mu })}^{k-1}\exp (-\frac{kx}{\mu }),$$

where *k* > 0 is the shape parameter, *µ* > 0 is the mean and Γ(*k*) is a gamma function. We consider two models to test if the route of infection (i.e. inoculation or contact) has an effect on the latent period. The first assumes that the route of infection has no effect, so inoculated and contact animals have their latent periods drawn from a common distribution, and the other has separate distributions for the two routes.

We assume that the presence of the pathogen in a sample taken from an animal means it is infectious and that the infectiousness of an animal is constant until it recovers or is removed. The force of infection exerted on each susceptible animal is given by:

2 $$\lambda (t)=\beta \frac{I(t)}{N(t)},$$

where *β* is the transmission parameter, *I*(*t*) is the number of infectious animals and *N*(*t*) is the total number of animals at time *t*. This formulation assumes frequency-dependent transmission and homogeneous mixing^7^. The basic reproduction number is simply given by the product of the transmission parameter and the mean infectious period (*μ*_*I*_):

3 $${R}_{0}=\beta \,{\mu }_{I}.$$

### Parameter inference

The generic model has five parameters to be inferred; the shape and mean for the gamma-distributed latent and infectious periods and the transmission parameter. As the infection times for contact animals are not directly observed, we include them in a data augmentation step. This also allows the unobserved latent periods to be inferred as nuisance parameters.

The likelihood is given by:

4 $$\begin{array}{rcl}L({\boldsymbol{\theta }},{{\bf{t}}}_{{\bf{I}}},{\bf{E}}) & = & \prod _{j\in {\rm{Uninfected}}}\exp ({\int }_{0}^{{t}_{{\rm{end}}}}-\lambda (\tau )\,d\tau )\\  &  & \times \prod _{j\in {\rm{Infected}}}\lambda ({t}_{I}^{(j)})\,\exp ({\int }_{0}^{{t}_{I}^{(j)}}-\lambda (\tau )\,d\tau )\\  &  & \times \prod _{j\in {\rm{Infected}}}{f}_{E}({E}_{j})\\  &  & \times \prod _{j\in {\rm{Censored}}}{\int }_{{t}_{lp}^{(j)}-{t}_{I}^{(j)}-{E}_{j}}^{\infty }{f}_{I}(\tau )\,{\rm{d}}\tau \\  &  & \times \prod _{j\in {\rm{Recovered}}}{\int }_{{t}_{lp}^{(j)}-{t}_{I}^{(j)}-{E}_{j}}^{{t}_{fn}^{(j)}-{t}_{I}^{(j)}-{E}_{j}}{f}_{I}(\tau )\,{\rm{d}}\tau ,\end{array}$$

where **θ** = {*k*_*E*_, *µ*_*E*_, *k*_*I*_, *µ*_*I*_, *β*} is the vector of model parameters, **t**_**I**_ is the vector of unobserved infection times and **E** is the vector of unobserved latent period durations. The first term is the probability that the surviving uninfected animals were not infected and the second term is the corresponding probability that each infected animal *j* became infected at time ${t}_{I}^{(j)}.$ The third term gives the contribution of the latent period *E*_*j*_ of animal *j*, with gamma-distributed probability density function *f*_*E*_(*E*) (see equation (1). The final two terms account for the gamma-distributed infectious periods of the censored animals, whether due to culling or experiment duration, and recovered animals, respectively, with probability distribution function *f*_*I*_(*I*) (see equation (1)).

Taking *t* = 0 to be the time at which inoculated animals are infected, the times of last negative and first positive samples provide constraints on each animal’s infection time and latent period:

5 $$0 < {t}_{I}^{(j)} < {t}_{fp}^{(j)},$$

and

6 $${t}_{ln}^{(j)} < {t}_{I}^{(j)}+{E}_{j} < {t}_{fp}^{(j)}.$$

Informative priors for all parameters can be constructed from separate experiments (see Case Studies below). If no suitable data are available, however, less-informative priors over biologically reasonable space can be used. The joint posteriors are sampled using an adaptive Metropolis algorithm^23,24^, with the scaling factor being adaptively adjusted to ensure an acceptance rate between 20% and 40%^25^. Four chains are run with mixing and convergence assessed visually and using Gelman-Rubin statistics, courtesy of the coda package^26^ in R^27^. Autocorrelation plots and effective sample sizes (the latter computed using the mcmcse package^28^ in R^27^) are used to choose appropriate length and thinning of the chains. For example, in the FMDV in pigs case study chains of 10,000,000 samples were run, with the preceding 10,000,000 samples discarded to allow for burn-in of the chain. Chains were subsequently thinned by taking every 1000th sample, with an effective sample size of around 2000. This took of the order of a few hours to run on a high-performance computing cluster.

All model code is written in C++ using the GNU Scientific Libraries^29^ for random number generation and the Eigen template library^30^ and is available online at github^31^. Equivalent Matlab code (version R2014b; The Mathworks, Inc.) for two case studies (FMDV in lambs and ASFV in pigs) is provided in the supplementary information, Appendix S1.

We compare the two models for the latent periods (i.e. independent of route of infection and dependent on route of infection) using the deviance information criterion (DIC)^32^ defined as:

$$D=-4\,\mathrm{log}\,\bar{L}({\boldsymbol{\theta }},{{\bf{t}}}_{{\bf{I}}},{\bf{E}})+2\,\mathrm{log}\,L(\bar{{\boldsymbol{\theta }}},{\bar{{\bf{t}}}}_{{\bf{I}}},\bar{{\bf{E}}}),$$

where *L* is the likelihood defined above (equation (4) and the bar denotes the (posterior) mean. Alternative definitions of the DIC are possible and these have been reviewed elsewhere^33^.

### Model checking

We first aim to demonstrate the validity of our methods and provide support for the inferences made. For this purpose, we used our SEIR model to simulate two transmission experiments, following the generic design described above with daily sampling. The first synthetic dataset used a 2 vs 2 design and the following parameters: *k*_*E*_ = 15.0, *μ*_*E*_ = 4.0, *k*_*I*_ = 5.0, *μ*_*I*_ = 8.0 and *β* = 0.15 (cf. FMDV in lambs). This yields *R*_0_ = 1.2, so that only a proportion of the contact animals would be expected to become infected. The second synthetic dataset used a 5 vs 5 design and the following parameters: *k*_*E*_ = 15.0, *μ*_*E*_ = 3.0 (inoculated), *k*_*E*_ = 10.0, *μ*_*E*_ = 6.0 (contact-challenged), *k*_*I*_ = 15.0, *μ*_*I*_ = 6.0 and *β* = 3.0 (cf. ASFV in pigs). This yields *R*_0_ = 18.0, so that almost all the contact animals would be expected to become infected. We extract the inputs our framework requires from the simulations and re-infer the model parameters, infection times for the contact animals and the latent periods for all infected animals (considering models with both common and separate latent periods). Wide, non-informative (i.e. uniform with range from 0 to 100) prior distributions were used for these analyses.

### Case studies

Here we review three published transmission experiments and describe the features that require specific adaptations to the generic framework. We fit our models to transmission experiments of FMDV between lambs^4^ and between pigs^11^ and of ASFV between pigs^5,16^.

#### FMDV in lambs

This is the simplest of the three examples presented here, following the generic design detailed above. A total of 24 lambs were used, housed in six separate rooms of four lambs each^4^. Two in each room were inoculated with the FMDV field isolate O/NET/2001, and after 24 hours, the remaining two naïve lambs were brought in to be challenged. This leads to a very small modification to equation (5), such that:

7 $$1 < {t}_{I}^{(j)} < {t}_{fp}^{(j)},$$

OPF swabs were taken daily up to day 14 post inoculation (dpi) and the presence of FMDV was determined by virus isolation. Three of the twelve contact-challenged lambs became infected, but their infection times and latent periods are unobserved. In addition, the latent periods for the inoculated lambs are only partially observed (i.e. known to a one-day window). Finally, six inoculated and two contact-challenged lambs were still positive at when the last OPF swab was taken (at 14 dpi), so their infectious periods are right-censored (see supplementary information, Data S1). The status of the nine contact-challenged lambs which were categorised as uninfected at the end of the experiment was confirmed by both a negative ELISA test (indicating no antibodies to viral non-structural proteins and, hence, no evidence of viral infection and replication) and negative probang samples taken at 28 to 30 dpi (indicating no persistent infection).

Informative priors were constructed for the model parameters using data from a similar but smaller transmission experiment in lambs^34^ (Supplementary Table S1). A second analysis was performed in which non-informative priors were used for all the model parameters (uniform with range 0 to 100).

#### FMDV in pigs

Two experiments^11^ were carried out together using the FMDV field isolate O/NET/2001, both of which were used to estimate parameters. In the first, six separate rooms each housed one inoculated pig with a naïve pig introduced one day post-inoculation (dpi). The second experiment linked two sets of challenges sequentially together. The infection started with four pigs being inoculated. After one day, these were moved in to a clean room housing five susceptible pigs, labelled C_1_. These C_1_ pigs were tested for presence of FMDV on a daily basis and, on the first positive result, all C_1_s were immediately moved to a new clean room housing five more susceptible pigs to be challenged, labelled C_2_. The whole procedure was carried out twice. All pigs involved developed clinical disease and six were culled on welfare grounds, though only after all transmissions had occurred.

As the first challenge occurred at one day post-inoculation, we use the same constraint on C_1_ infection times as in equation (7). We also have the infection times for the C_2_ pigs being constrained by the day the C_1_ pigs are moved:

8 $$\mathop{\min }\limits_{j\in {C}_{1}}({t}_{fp}^{(j)}) < {t}_{I}^{(j)} < {t}_{fp}^{(j)},$$

OPF swabs were taken daily for all pigs in the first experiment and the C_1_ and C_2_ pigs from the second and virus isolations were performed to test for presence of FMDV. As the time-series data for the inoculated pigs in the second experiment was not available, we simply treat these as the infectious source for the C_1_ pigs for the entire duration of contact and make no inferences on the inoculated pigs’ latent or infectious periods. In one of the one-to-one transmission experiments, the inoculated pig did not become infectious, nor did the contact pig become infected; this experiment was excluded from the analysis. The remaining 25 contact challenged pigs became infected and all were included in the analysis.

To quantify the effectiveness of vaccination, both of these experiments were repeated with all contact-challenged pigs being vaccinated at −14 dpi^11^. As the infection chain was started by inoculating unvaccinated pigs, we fit our models to data from the second experiment and only infer parameters for the vaccinated pigs.

In all experiments, the latent periods for the inoculated pigs are only partially observed, the infection times and latent periods for the contact-challenge pigs are unobserved and four of the unvaccinated contact-challenged pigs were euthanized on welfare grounds and, hence, observations for their infectious periods are right-censored (see supplementary information, Data S2).

Informative priors for the model parameters were constructed using another transmission experiment in pigs^35^ (Supplementary Table S1). A second analysis was performed in which non-informative priors were used for all the model parameters (uniform with range 0 to 100).

#### ASFV in pigs

Here a set of four transmission experiments were carried out to quantify transmission of the highly virulent Georgia 2007/1 strain of ASFV^5,16^, currently circulating in Eastern Europe. Groups of five, four, four or three inoculated pigs (for rooms A–D, respectively) were in direct contact with an equal number of susceptible pigs. For rooms B and C, a further group of four susceptible pigs was housed in a separate pen within the same room, allowing indirect contact only. A total of 40 pigs were used in these experiments; 16 were inoculated, 16 in direct contact and 8 in indirect contact. All pigs developed clinical disease and were culled on welfare grounds.

To account for the second pen, we introduce a between-pen transmission term and infer parameters for both within- and between-pen transmission. The force of infection exerted on susceptible animals in each pen is given by:

9 $$\begin{array}{c}{\lambda }_{1}(t)={\beta }_{W}\frac{{I}_{1}(t)}{{N}_{1}(t)}+{\beta }_{B}\frac{{I}_{2}(t)}{{N}_{1}(t)+{N}_{2}(t)},\\ {\lambda }_{2}(t)={\beta }_{W}\frac{{I}_{2}(t)}{{N}_{2}(t)}+{\beta }_{B}\frac{{I}_{1}(t)}{{N}_{1}(t)+{N}_{2}(t)},\end{array}$$

where *β*_*W*_ and *β*_*B*_ are the within- and between-pen transmission parameters, *I*_*i*_(*t*) and *N*_*i*_(*t*) are the number of infectious and total number of pigs in pen *i* at time *t*. The likelihood in equation (4) is modified to account for the pen in which each animal is housed.

Blood samples were taken from all pigs, starting at three days post inoculation (dpi) of the seed pigs and every two days thereafter, and were tested for presence of ASFV by virus isolation (see supplementary information, Data S3). In this experiment, the latent periods of the inoculated pigs are only partially observed (the first samples for some inoculated pigs were positive), the infection times and latent periods for the contact-challenged pigs are unobserved and, finally, all infected animals were euthanized on welfare grounds while still positive, so all infectious periods are right-censored.

Informative priors for each of the parameters were constructed based on previous transmission experiments^36^ and outbreaks of the Georgia 2007/1 strain in the Russian Federation^37^ (Supplementary Table S1). A second analysis was performed in which non-informative priors (uniform with range 0 to 100) were used for all the model parameters, except the infectious period parameters. For these two parameters informative priors had to be used because all infectious periods were right-censored.

### Data availability

All data analysed as part of this study are available from the original publications^4,11,16^. They are provided in the supporting information (Data S1–S3) in the format they were used in the analysis.

## Results

### Model checking

In the synthetic 2 vs 2 experiment, 21/50 contact animals became infected. The inferences on infection times and latent periods are shown in Supplementary Fig. S1, where we see the 95% highest-posterior density interval (HPDI) for each infection time contains the known value. The model parameters all lie within the 95% HPDI with posterior medians close to the known values, while the deviations of the inferred infection times are centred on zero (Supplementary Fig. S2).

The synthetic 5 vs 5 experiment was generated assuming separate latent periods for inoculated and contact-infected animals and with a much higher *R*_0_. In this case, all 50 contacts became infected and all actual infection times are within the 95% HPDI of our inferences (Supplementary Fig. S3). As with the 2 vs 2 synthetic dataset, the 95% HPDI contain the parameter values used to generate the data and the deviations of the infection times show no obvious signs of bias (Supplementary Fig. S4). Furthermore, the model with separate latent periods was strongly preferred to the model with common latent periods (DIC = 836.4 for the model with separate latent periods compared with DIC = 900.5 for the model with a common latent period).

### FMDV in lambs

**Figure 2:**
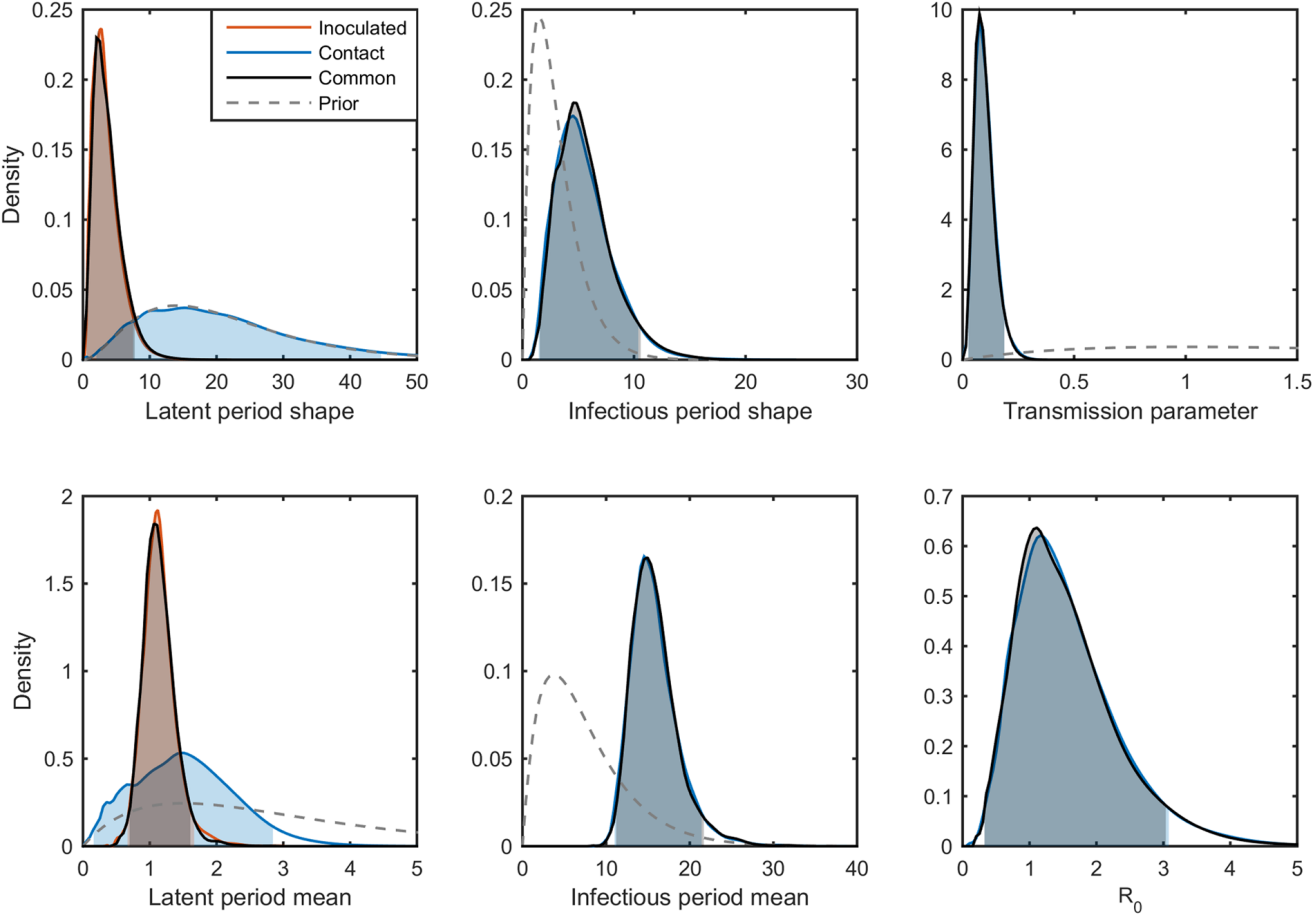
Epidemiological parameters for foot-and-mouth disease virus (FMDV) in lambs. Marginal posterior distributions for each parameter inferred from the FMDV transmission experiments between lambs. The latent period shape and mean posteriors are plotted in blue for in-contact lambs and in orange for inoculated. The model with a combined latent period distribution is plotted in grey. The shaded areas indicate the 95% highest posterior density intervals and priors are plotted as grey dashed lines.

Based on the DIC we found no significant evidence for a difference in latent periods between intranasally inoculated and contact-infected lambs (DIC = 94.5 for the model with separate latent periods compared with DIC = 95.6 for the model with a common latent period). In the model with separate latent periods, inoculation with the virus resulted in shorter latent periods than contact infection, with a difference in mean duration of 0.37 days. The marginal posteriors for the model with common latent period are summarised in Table 1 and the posterior distributions for both models are plotted in Fig. 2. We see that lambs are infectious for a long time, but at a low rate of transmission. The resulting estimate (posterior mean) for *R*_0_ is 1.43, with the 95% HPDI including the threshold value of *R*_0_ = 1.

**Table 1:** Posterior median and 95% highest-posterior density intervals (HPDI) for gamma-distributed latent and infectious period parameters, transmission parameter and basic reproduction number for three transmission experiments.

		Latent period^†^				Infectious period		Transmission parameter		*R*_0_	
		Contact		Inoculated							
		Shape	Mean	Shape	Mean	Shape	Mean	Within-pen	Between-pen	Within-pen	Between-pen
FMDV in lambs	mean	3.31	1.12	—	—	5.22	15.4	0.09	—	1.43	—
	95% HPDI	(0.53, 7.74)	(0.70, 1.61)	—	—	(1.56, 10.6)	(11.2, 21.7)	(0.03, 0.19)	—	(0.33, 3.04)	—
FMDV in pigs (unvaccinated)	mean	1.35	0.14	1.39	0.97	14.14	5.70	1.51	—	8.54	—
	95% HPDI	(0.22, 3.65)	(0.01, 0.33)	(0.18, 3.39)	(0.40, 1.67)	(6.86, 22.1)	(5.12, 6.33)	(0.75, 2.55)	—	(4.28, 14.9)	—
FMDV in pigs (vaccinated)	mean	1.44	0.27	—	—	5.55	4.74	0.36	—	1.70	—
	95% HPDI	(0.24, 3.79)	(0.03, 0.65)	—	—	(2.38, 9.66)	(3.82, 5.85)	(0.16, 0.59)	—	(0.73, 2.91)	—
ASFV in pigs	mean	19.2	6.08	12.0	2.80	22.7	9.15	2.62	0.99	24.1	9.17
	95% HPDI	(6.77, 36.7)	(4.94, 7.19)	(2.69, 27.6)	(2.28, 3.31)	(4.77, 53.4)	(6.67, 12.3)	(0.96, 5.61)	(0.31, 1.98)	(7.34, 54.2)	(2.67, 19.2)

^†^For FMDV in lambs there was no significant difference in latent period between inoculated and contact-infected animals.

**Figure 3:**
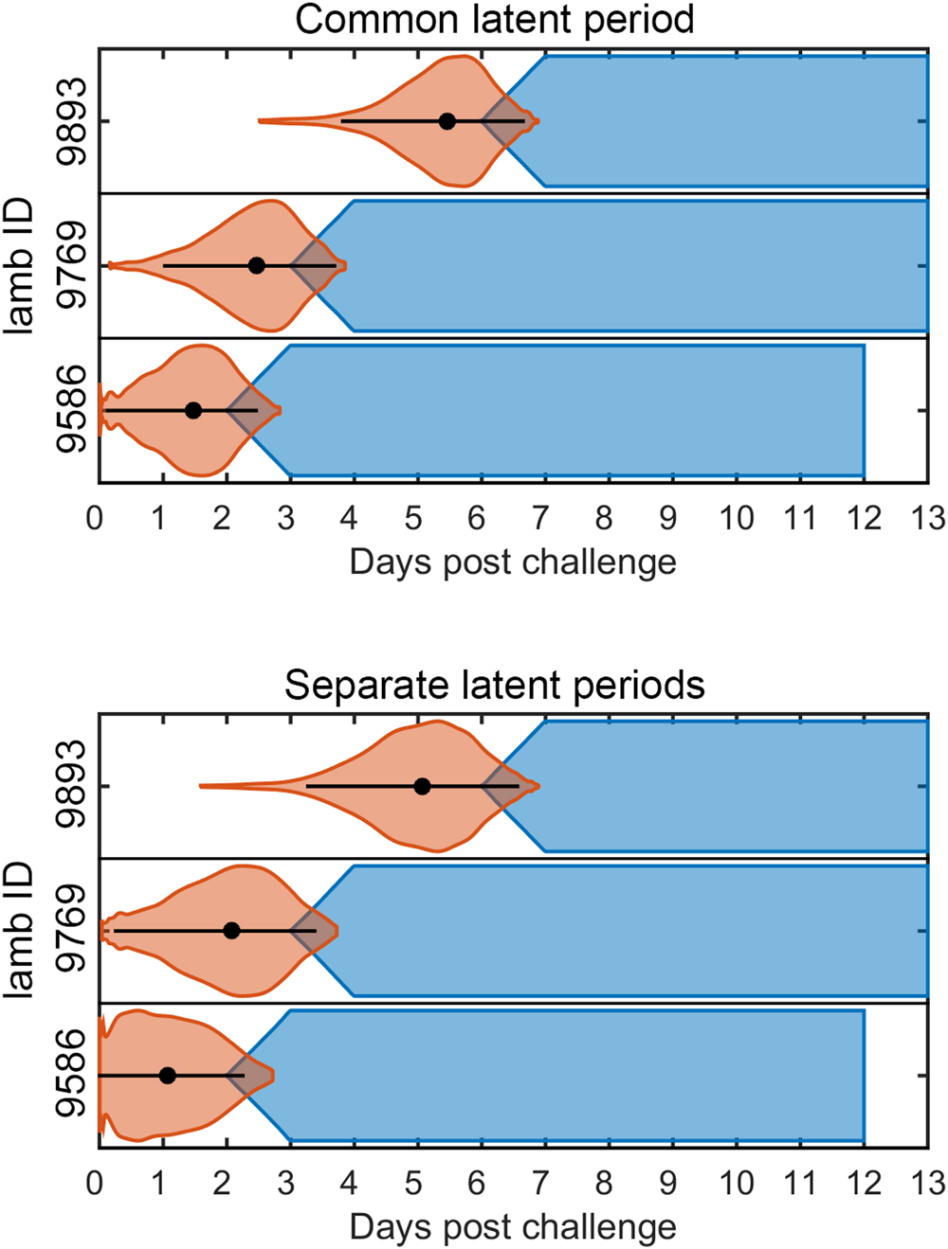
Inferred infection times for the FMDV in lambs transmission experiment. Orange violin plots showing the densities of the inferred infection times for the contact lambs assuming a common latent period (top panel) or separate latent periods (bottom panel) for inoculated and contact-infected lambs. The black circles and bars denote the posterior median and the 95% highest posterior density interval. The inferred cumulative probability of animals being infectious at each time point are shown in blue.

The inferred infection times for the three (out of 12) contact lambs which became infected are shown in Fig. 3. The two lambs (9893 and 9769) are from the same group (group D) and the infection times suggest that one contact lamb (9769) could have acted as the source of infection for the other (9893).

Using non-informative priors did not greatly affect the posterior estimates for any of the model parameters, except for the shape parameter for the infectious period (*k*_*I*_) which was much higher (posterior mean: 22.9; 95% HPDI: 4.8–56.1) (cf. Table 1).

### FMDV in pigs

Here the DIC strongly favoured the model with separate latent period distributions for the inoculated and contact-transmitted pigs (DIC = 78.0 compared with DIC = 100.2 for the model with a common latent periods). We found the latent period for contact-infection to be very short with a mean of 0.14 days, which is in agreement with previous observations^35,38^. The pigs inoculated in the bulb of the heel, however, have a longer latent period with a mean of 0.97 days.

**Figure 4:**
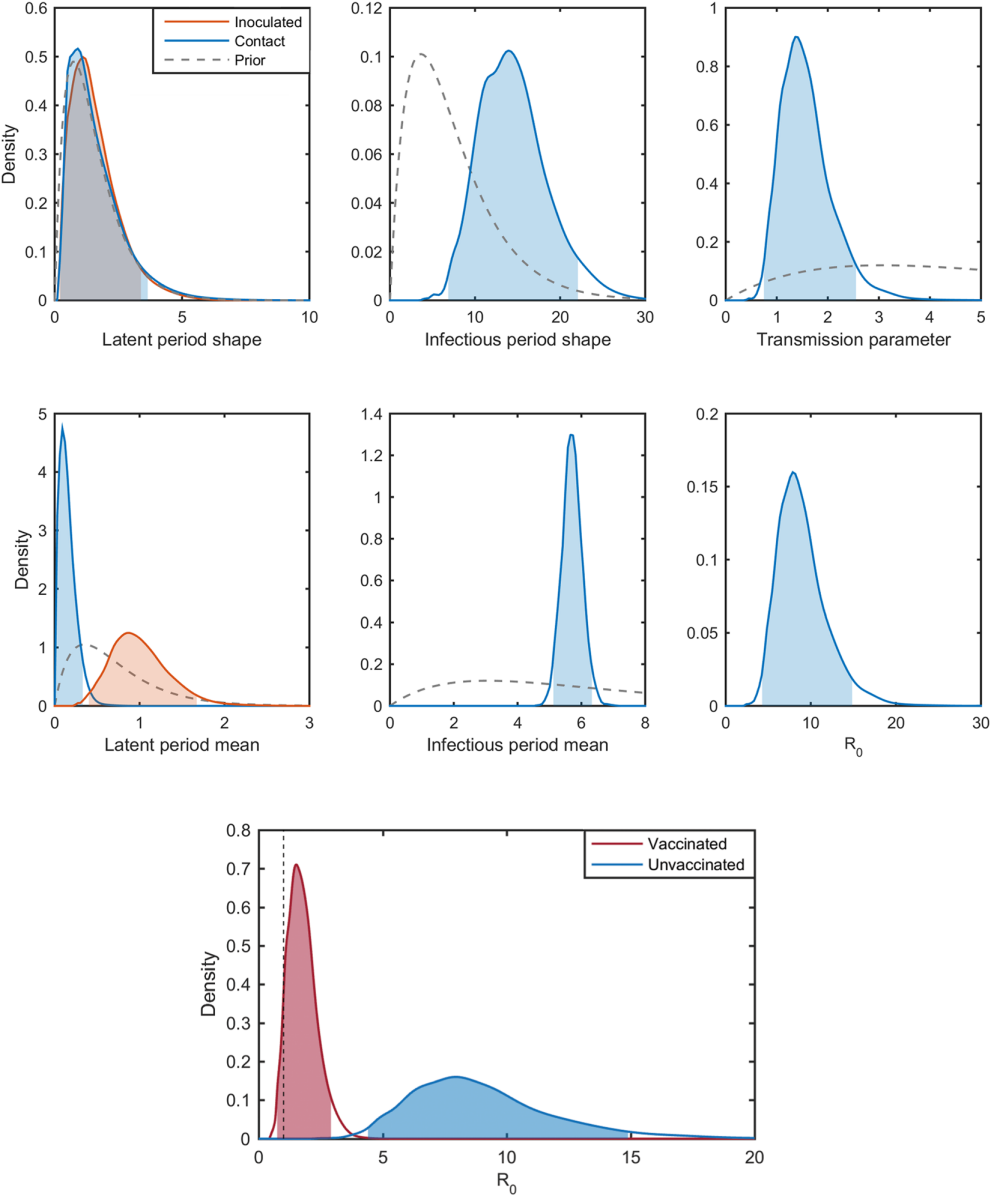
Epidemiological parameters for foot-and-mouth disease virus (FMDV) in pigs. Marginal posterior distributions for each parameters inferred from the FMDV transmission experiments between unvaccinated pigs (top panel). The latent period shape and mean posteriors are plotted in blue for in-contact pigs and in orange for inoculated. The shaded areas indicate the 95% highest posterior density intervals and priors are plotted as grey dashed lines. Posterior density plots of *R*_0_ for vaccinated pigs in red and unvaccinated pigs in blue (bottom panel), with the shaded areas representing the 95% highest-posterior density interval. The dashed black line indicates the threshold value of *R*_0_ = 1.

**Figure 5:**
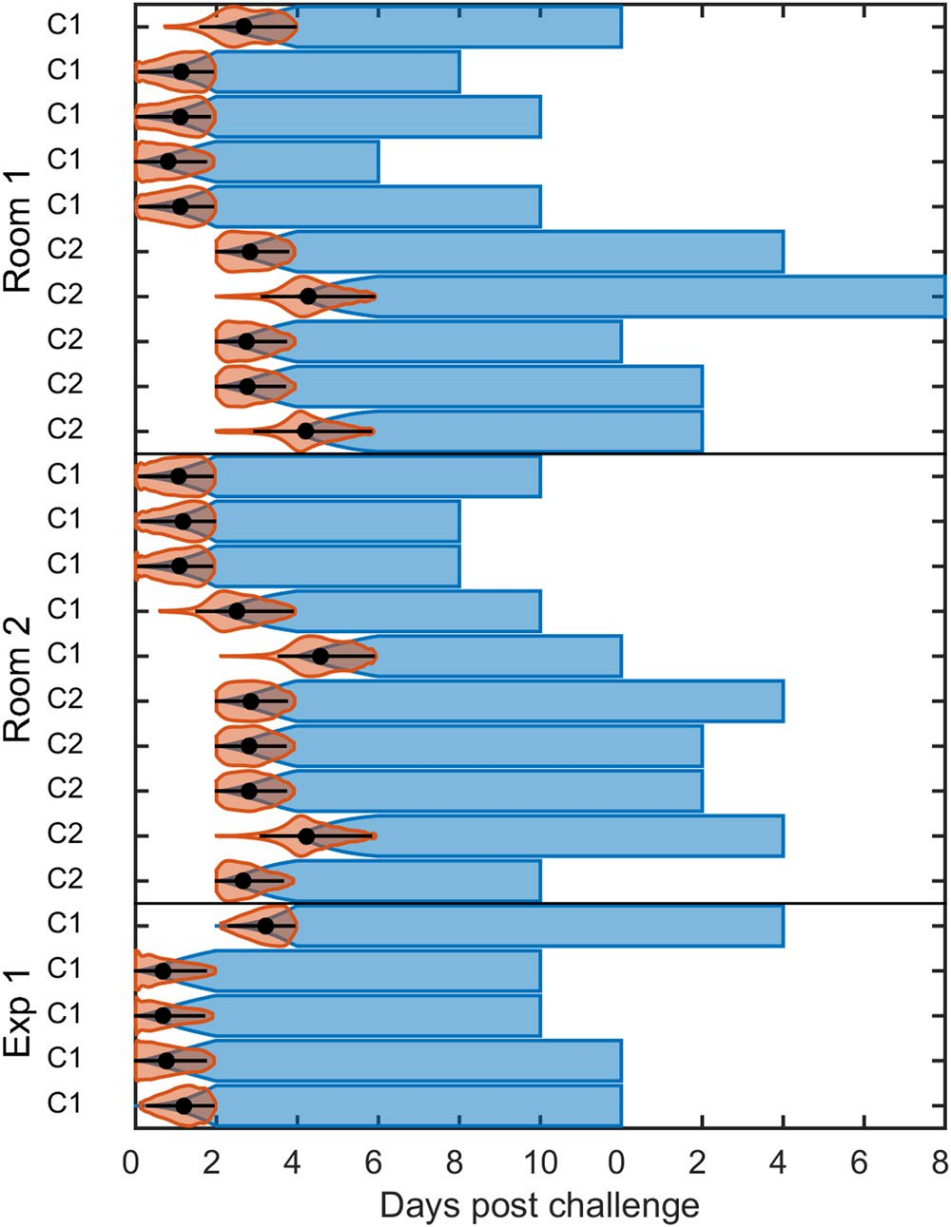
Inferred infection times for the FMDV in pigs transmission experiments. Orange violin plots showing the densities of the inferred infection times for the unvaccinated contact pigs. The black circles and bars denote the posterior median and the 95% highest posterior density interval. The inferred cumulative probability of animals being infectious at each time point are shown in blue.

As expected, given that all the unvaccinated pigs in these experiments became infected, our estimate of *R*_0_ = 8.54 (4.41, 14.9) is large and excludes the threshold value of *R*_0_ = 1. The marginal posterior distributions for the model parameters are plotted in Fig. 4 and summarised in Table 1. The inferred infection times are shown in Fig. 5. The short latent periods and rapid transmission make conclusions about who infected whom difficult to draw. However, it is clear that some of the C_1_ pigs in both experiments were infected by other C_1_ pigs rather than the inoculated pigs to which they were initially exposed (Fig. 5). Similarly, some of the C_2_ pigs could have been infected by other C_2_ pigs rather than the C_1_ pigs.

When non-informative priors were used, the posterior estimates for the mean latent period (for both inoculated and contact challenged pigs), the mean infectious period and the transmission parameter did not differ greatly from those obtained using informative priors. However, the shape parameters for the distributions were much higher (posterior means (95% HPDI) of 48.6 (2.2–97.3), 5.5 (0.6–16.8) and 48.5 (9.4–95.9) for the latent period for contact challenged pigs, for the latent period for inoculated pigs and for the infectious period, respectively) (cf. Table 1).

For vaccinated pigs, we estimated *R*_0_ to be 1.7 (0.74, 2.92). The effect of vaccination on *R*_0_ is clearly shown in Fig. 4, with a difference in posterior medians of 6.84 and non-overlapping HPDIs. The model parameters for the vaccinated pigs are plotted in Supplementary Fig. S5 and summarised in Table 1. The inferred infection times are plotted in Supplementary Fig. S6. A similar pattern of who infected whom is seen as that in the unvaccinated pigs (cf. Figure 5), but the reduced rate of spread provides greater evidence for transmission within groups of pigs (i.e. C_1_ to C_1_ or C_2_ to C_2_).

### ASFV in pigs

**Figure 6:**
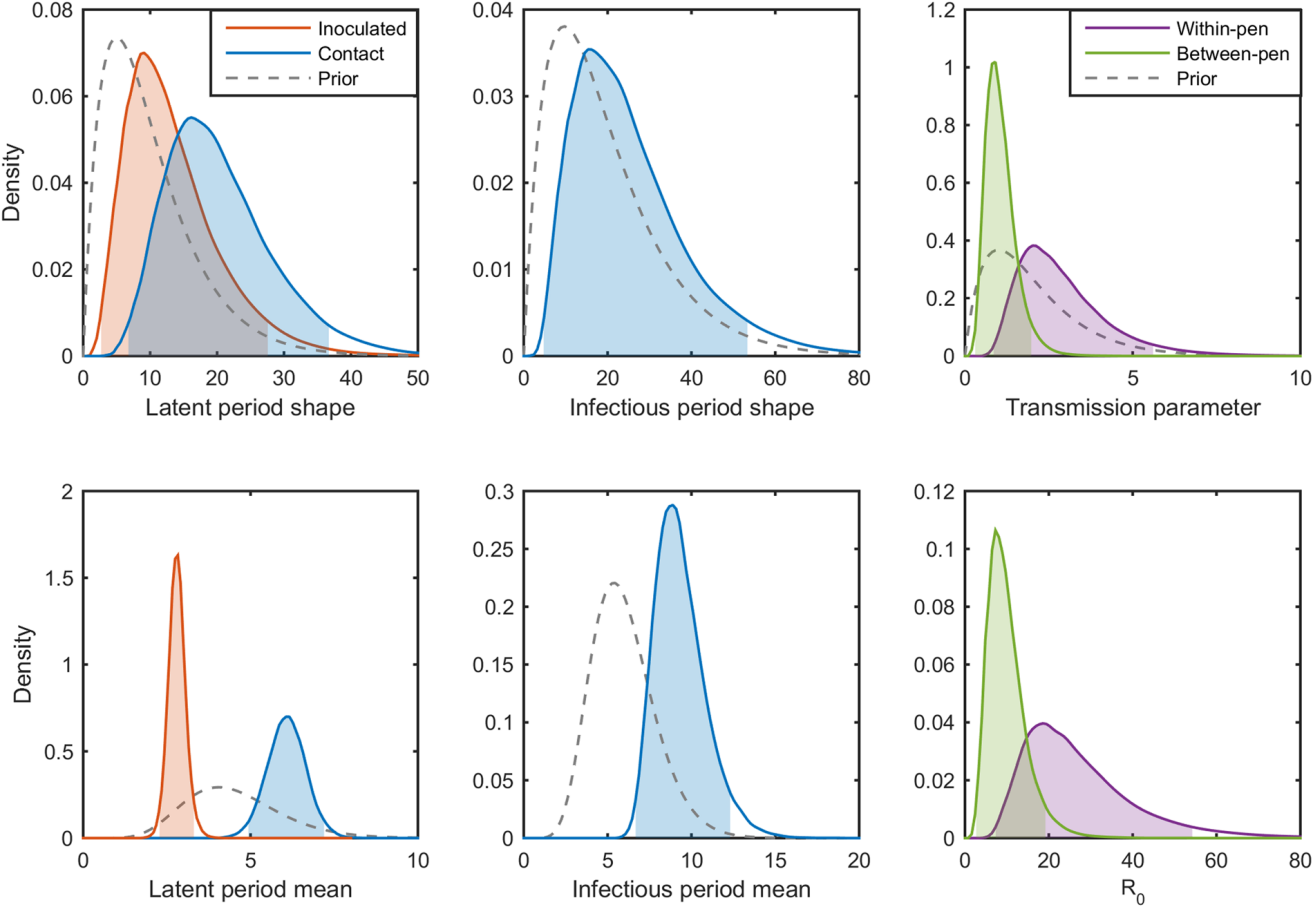
Epidemiological parameters for African swine fever virus (ASFV) in pigs. Marginal posterior distributions for each parameter inferred from the ASFV transmission experiments. The latent period shape and mean posteriors are plotted in blue for in-contact lambs and in orange for inoculated. The transmission parameter and *R*_0_ posteriors are plotted in purple for within-pen transmission and in green for between-pen transmission. The shaded areas indicate the 95% highest posterior density intervals and priors are plotted as grey dashed lines.

The model with separate latent periods for the inoculated and contact-infected pigs was again preferred (DIC = 215.6 compared with DIC = 248.9 for the model with a common latent period distributions). The difference in latent periods of 4.9 days is clearly substantial, as can be seen in Fig. 6. Transmission parameters for direct and indirect contact were estimated, showing that transmission within pens occurs at a much higher rate than between pens. Our estimates of *R*_0_ for both routes of transmission are well above the critical threshold of *R*_0_ = 1, suggesting that limiting direct contact would not be enough to prevent an outbreak (Table 1).

**Figure 7:**
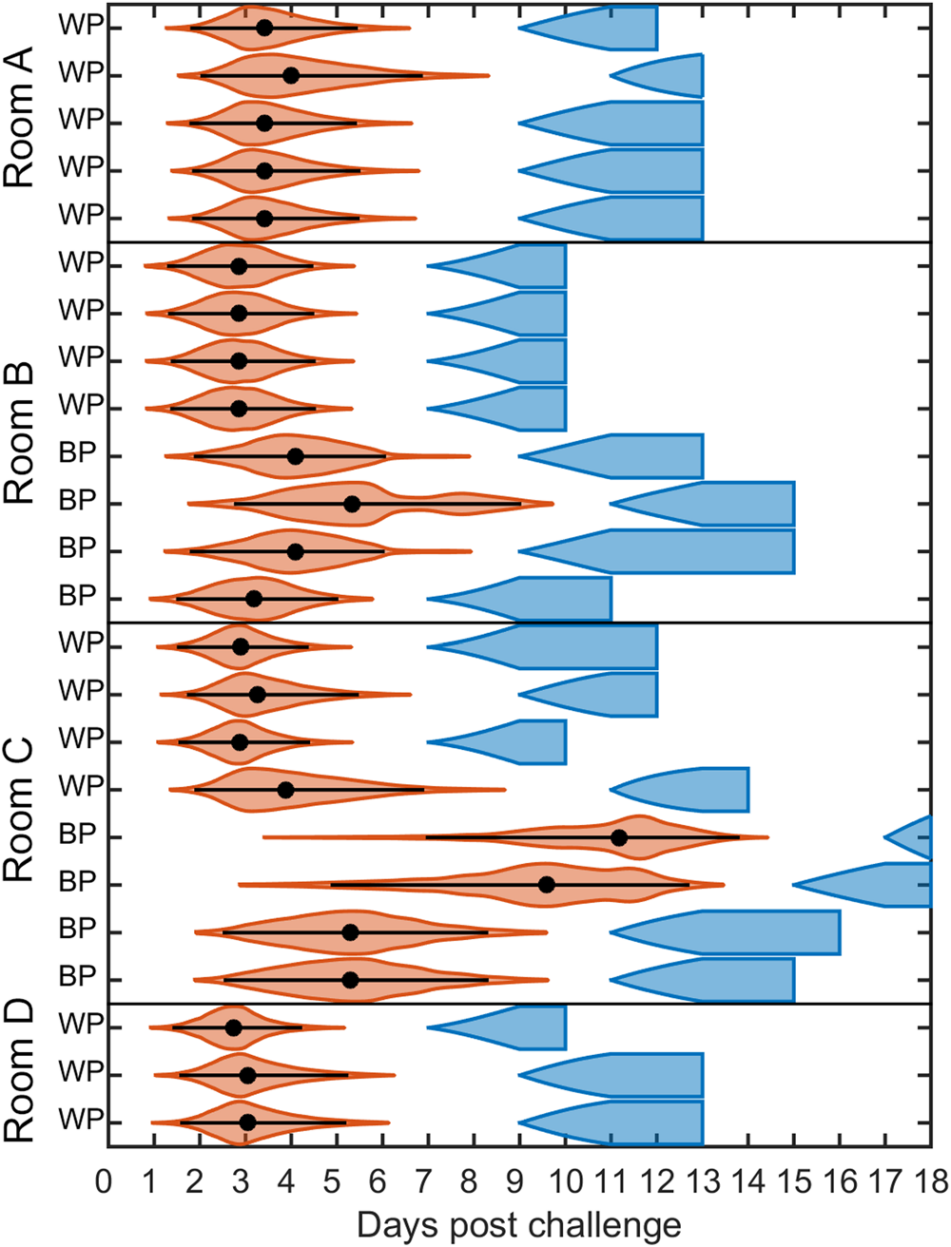
Inferred infection times for the ASFV in pigs transmission experiment. Orange violin plots showing the densities of the inferred infection times for the contact pigs (WP: within-pen contacts; BP: between-pen contacts). The black circles and bars denote the posterior median and 95% highest posterior density interval. The inferred cumulative probability of animals being infectious at each time point are shown in blue.

The infection times for each in-contact pig are shown in Fig. 7. For the two experiments in which there were only within-pen contacts the in-contact pigs were infected by the inoculated pigs. For the two experiments in which there were both within- and between-pen contacts (rooms B and C), the within-pen contacts were typically infected by the inoculated pigs. In room B the between-pen contacts were also most likely infected by the inoculated pigs, though it is possible one was infected by the within-pen contacts. By contrast, in room C the infection times suggest that two of the between-pen contacts were infected by inoculated pigs, while the remaining two were infected by the within-pen contacts.

Using non-informative priors for the model parameters did not greatly affect the posterior estimates for the mean latent periods (in both inoculated and contact animals). However, the shape parameter for the latent periods were much higher (contact challenged pigs: 36.3 (14.7–64.7); inoculated pigs: 59.7 (12.2–98.1)) (cf. Table 1). In addition, the estimates for the transmission parameters were also higher, markedly so for within-pen transmission (within-pen: 33.2 (4.0–77.1); between-pen: 1.4 (0.5–2.9)) (cf. Table 1).

## Discussion

In this study we have used a Bayesian framework to infer key epidemiological parameters from transmission experiment data. We detailed a generic form of the model that explicitly accounts for many of the issues inherent to such data, namely that the infection times and latent and infectious periods are not directly observed and that the infectious periods may also be censored. We also showed the flexibility of this approach by adapting the model to three different experimental designs.

To check the inferences made using our Bayesian framework, especially regarding the unobserved infection times and latent periods, we fit the model to synthetic data. This has the advantage that we have complete information when generating the synthetic data set, allowing us to compare the inferred infection times with those known from the simulations. For two scenarios we demonstrated that our methods do indeed infer infection times correctly (i.e. the actual infection times lie in the 95% HPDI) (Supplementary Figs S1 and S3) and without any obvious biases (Supplementary Figs S2 and S4), giving us some confidence that the infection times we infer from the experimental data are realistic. In addition, our methods also allow objective identification of a difference in latent periods between inoculated and contact-infected animals when one is present.

In the studies on FMDV transmission between lambs, six of the inoculated lambs’ OPF tested positive for virus at the end of the experiment, meaning transmission could still occur, although with very low probability^39^. This would potentially violate the assumption that the final size of the epidemic had been reached. As a result *R*_0_ would be underestimated by the FS method (*R*_0_ = 1.14) and overestimated by the GLM method (*R*_0_ = 2.22)^4^. Indeed, our estimate is somewhere between these values: posterior median *R*_0_ = 1.43. As all animals in the experiments using FMDV or ASFV in pigs became infected, the FS method does not provide usable estimates of *R*_0_, with *R*_0_ = ∞ being published in the previous analysis of the data for FMDV in pigs^11^. In addition, the FS method was unable to identify a significant impact of vaccination on FMDV in pigs^11^, whereas we showed a significant reduction in *R*_0_ for vaccinated compared with unvaccinated pigs (Table 1; Fig. 4). This helps demonstrate a strength of the Bayesian approach in that we can combine data from independent sources and construct informative priors, restricting inferences to biologically reasonable space. Furthermore, these results can be readily updated with data from any future experiments as they become available.

The importance of accounting for censored infectious periods is made particularly clear with the study on ASFV, where all pigs had to be euthanized on welfare grounds. This means it is not possible to infer infectious periods from the experimental results alone. In the previous analysis they were assumed to be normally distributed (mean ± standard deviation) for either 4.50 ± 0.75 days or 8.50 ± 2.75 days^16^. By contrast, our models were able to directly infer the parameters for gamma distributed infectious periods, finding the mean duration (posterior median and 95% HPDI) to be 9.15 (6.68, 12.3) days.

The GLM method assumes latent periods to be fixed and known^12^. In the ASFV transmission experiments, three model variants with latent periods of 3, 4 and 5 days were fit^16^. The model assuming a latent period of 5 days was favoured by Akaike’s information criterion, whereas our equivalent model estimated the mean latent period to be 3.3 days. However, we found strong evidence suggesting that the infection route has a large effect, specifically that the mean latent period (posterior median and 95% HPDI) for intramuscular inoculation was 2.80 (2.30, 3.33) days and for contact-infection was 6.08 (4.95, 7.21) days. A difference in latent periods for inoculated and contact-infected animals was also observed in both FMDV experiments, highlighting the need to account for the transmission route and to infer latent periods directly. The size of the effect may depend upon the site of inoculation. The most marked difference we observed came in the FMDV in pigs experiments, where the inoculation was to the bulb of the heel. By contrast, the intranasal inoculation used in the FMDV in lambs experiments had a much reduced, and statistically non-significant, effect. Any differences are, of course, likely to be virus, dose and species specific, but should certainly be considered when analysing future experiments.

Although previous analyses of transmission experiments have included the unobserved infection times^14,15^, they have typically been treated simply as nuisance parameters. Yet the inferred infection times, when combined with information on which animals were infectious at which times, can provide insights into who infected whom (Figs 3, 5 and 7). This is particularly evident in the FMDV in lambs and in the ASFV in pigs experiment. In the latter, for example, we can identify two phases of infection in the rooms (B and C) including both within- and between-pen contacts, though this is clearest for room C (Fig. 7). In the first wave, the inoculated pigs infect the within-pen contact animals and, in the second wave, the within-pen contact animals infect the between-pen contacts. Linking the inferences about infection times with inferences from viral sequencing of samples from the infected animals^40,41^ has the potential to help resolve transmission pathways or, alternatively, to provide a separate confirmation of who infected whom.

In addition to providing insights into who infected whom, the inferred infection times can also help inform transmission mechanisms. For example, in the FMDV in lambs experiment the times at which the contact animals were inferred to become infected (Fig. 3) correlate with the peak viral titre in OPF for the lambs that were the probable source of infection (see Table 2 in ref.^4^). For example, the contact lamb (9769) which may have transmitted to its room-mate (9893) (Fig. 3) had a higher viral titre than either of the inoculated lambs in the room, providing evidence that this was the more likely route of infection. Finally, the infection times indicate that transmission occurred before the onset of clinical signs in the donor lambs, which is consistent with observations from the field during the 2001 epidemic in the United Kingdom^42^. This contrast with cattle, where a majority of infectiousness occurs after clinical onset^43^. Although the patterns of who infected whom are less clear in the FMDV in pigs experiments, there is still some evidence that transmission is correlated with peak viral titre in OPF (see Table 3 in ref.^11^; cf. Figure 5 and S6).

The issue with being unable to observe infection times stems directly from the experimental design, where the emphasis has been on estimating total transmission rates in groups of animals over extended periods of time. An alternative approach, used in transmission experiments of FMDV in cattle^43^, is to focus on individual transmission events in a series of short challenges at specific time-points post infection. With a range of samples being taken regularly, it is possible to quantify the relationship between the within-host dynamics and the actual transmission potential^44^. In the specific case of FMDV in cattle, the use of virus detection in nasal fluid, blood or OPF as proxies for infectiousness, instead of the direct occurrence of transmission itself, led to very different estimates of latent and infectious periods^43^. The benefits of this more complicated design must be weighed against the increased labour and logistical issues involved with using and moving many more animals. The methodology we present in this paper goes some way to reduce the gap in information that comes with the cheaper and easier experimental designs.

Our models have only used data on whether the samples tested positive or negative for virus presence, assuming that the animals all have constant and equal infectiousness throughout their infectious period. As there are often data available quantifying the viral load in each animal^4,11^, it would be possible to design models to relax this assumption. One approach kept each animal’s infectiousness constant but with a power-law dependency upon the peak level of viraemia^13^. A time-dependence could also be introduced, for example by assuming some functional form with parameter(s) to be estimated^45^. We have opted against this additional complexity in order to limit the computational expense of within-farm outbreak simulations, which can be particularly important for larger scale models^46^.

The proper quantification of the uncertainty in epidemiological parameters is crucial for further modelling work, particularly when scaling upwards for larger-scale stochastic simulations of disease outbreaks. Our work has shown that some of the assumptions on latent periods made in existing models of national-scale epidemics, for example, fixed at 4 or 5 days for FMDV in sheep and cattle^47–49^, can be improved upon. Explicit modelling of the within-farm dynamics, although more computationally-intensive, can replace such assumptions and provide more biologically robust conclusions.

One of the main goals of epidemic modelling is to inform policy on optimum control strategies^47^ and an important consideration with any such strategy would be vaccination^48^. It is extremely important to know how vaccinated animals respond to challenge, i.e. the level of protection provided by the vaccine, the duration of any protection, and any effects on onward transmission should a vaccinated animal become infected. Vaccination trials often follow the same procedures as we have described^4,8,9,11^, and so our model framework can be easily adapted to explore such data and provide inferences on the same epidemiological parameters required for modelling.

## Electronic supplementary material

Supplementary information
