# Supplementary material for: Bayesian inference of epidemiological parameters from transmission experiments
Source: Sci Rep. 2017 Dec 1;7:16774. doi: 10.1038/s41598-017-17174-8 (PMC5711876; doi:10.1038/s41598-017-17174-8)
Supplement: Supplementary file 1 — Supplementary information [file 41598_2017_17174_MOESM1_ESM.pdf]

Supplementary information

Bayesian inference of epidemiological parameters from transmission experiments

Ben Hu[1,2], Jose Gonzales[3] & Simon Gubbins[1,*]

[1] *The Pirbright Institute, Ash Road, Pirbright, Surrey GU24 0NF, U.K.*

[2] *Centre for Complexity Science, University of Warwick, Coventry CV4 7AL, U.K.*

[3] *Wageningen BioVeterinary Research, Houtribweg 39, 8221 RA, Lelystad, The Netherlands*

* Corresponding author (email: simon.gubbins@pirbright.ac.uk; Tel. +44 (0) 1483 232441; Fax. +44 (0) 1483 232448)

Table S1. Gamma prior distributions* used in the analysis of three transmission experiments.

experiment	latent period		infectious period		transmission parameter
	shape	mean	shape	mean	
FMDV in lambs†	Gamma(3,21)	Gamma(2,3)	Gamma(2,3)	Gamma(2,7.5)	Gamma(2,2)
FMDV in pigs‡	Gamma(2,1.5)	Gamma(2,0.7)	Gamma(2,7.3)	Gamma(2,6.1)	Gamma(2,6.1)
ASFV in pigs††	Gamma(2,10)	Gamma(10,4.5)	Gamma(2,19.3)	Gamma(10,6.0)	Gamma(2,2)

* Parameters for the gamma distribution are the shape and mean, respectively.

† Priors were constructed from a similar but smaller transmission experiment in lambs[1]. Specifically, gamma distributions were fitted to the virus shedding data to estimate the latent and infectious period parameters. A mildly-informative prior was selected for the transmission parameter.

‡ Priors were constructed from another transmission experiment in pigs[2]. Specifically, gamma distributions were fitted to the virus shedding data to estimate the latent and infectious period parameters, while the authors' estimate for the transmission parameter was used as the prior mean.

†† Priors were constructed for the latent and infectious period parameters based on previous transmission experiments with a different strain[3]. A gamma prior for the within- and between-pen transmission parameters was constructed from estimates obtained from outbreaks of the Georgia 2007/1 strain in the Russian Federation[4], which were also consistent with transmission experiments using different strains[3].

References for Table S1

1. Parida, S. *et al*. Emergency vaccination of sheep against foot-and-mouth disease: significance and detection of subsequent sub-clinical infection. *Vaccine* **26**, 3469-3479 (2008).
2. Eblé, P., De Koeijer, A., Bouma, A., Stegeman, A. & Dekker, A. Quantification of within-and between-pen transmission of foot-and-mouth disease virus in pigs. *Vet. Research* **37**, 647-654 (2006).
3. de Carvalho Ferreira. H. *et al*. Transmission rate of African swine fever virus under experimental conditions. *Vet. Microbiol.* **165**, 296-304 (2013).
4. Gulenkin, V., Korennoy, F., Karaulov, A. & Dudnikov, S. Cartographical analysis of African swine fever outbreaks in the territory of the Russian Federation and computer modeling of the basic reproduction ratio. *Prev. Vet. Med.* **102**, 167-174 (2011).

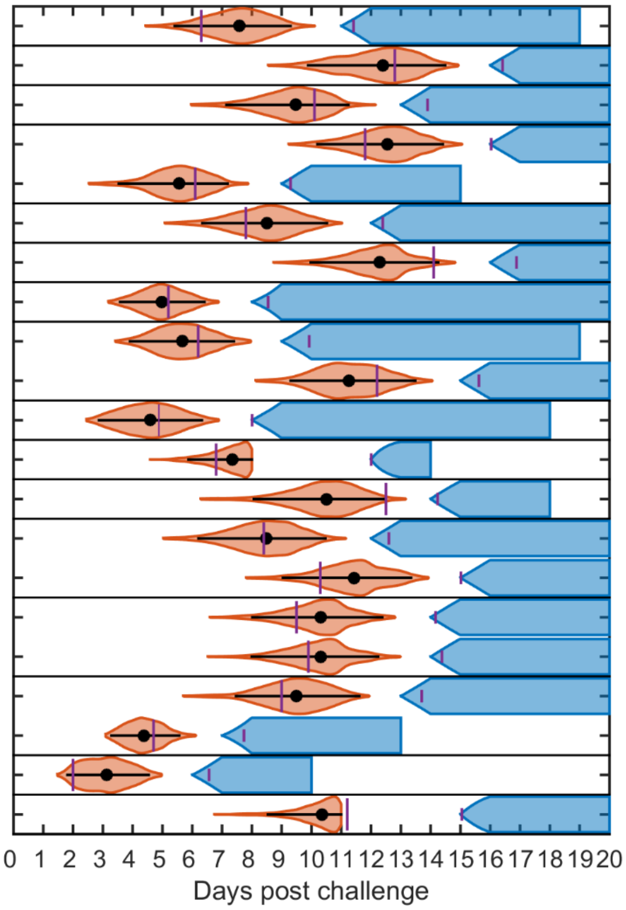

Figure S1. Inferred infection times for a synthetic 2 vs 2 transmission experiment. Orange violin plots show the posterior densities of the inferred infection times and the blue shapes represent the cumulative probability of each lamb being infectious at each time point. The black circles and bars denote the posterior median and the 95% highest posterior density interval. The longer purple lines mark the known infection times and the shorter purple lines indicate the start of the infectious period.

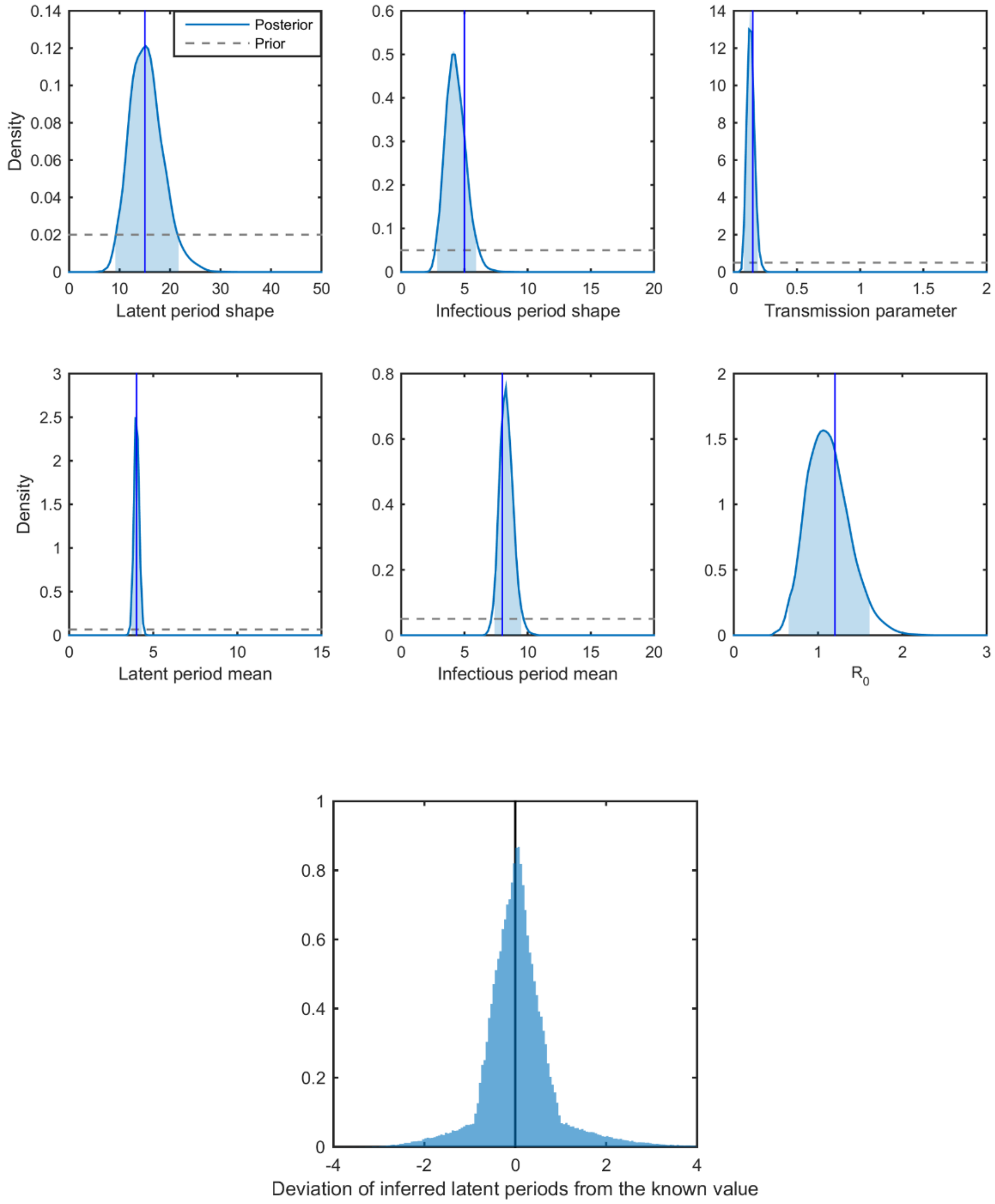

Figure S2. Inferred epidemiological parameters for a synthetic 2 vs 2 transmission experiment. Marginal posterior distributions for the parameters from the 2 vs. 2 synthetic dataset, with values used in the simulations indicated by vertical lines (top panels). The shaded area indicates the 95% highest posterior density interval and priors are plotted as grey dashed lines. Histogram of deviations of the inferred infection times (bottom panel).

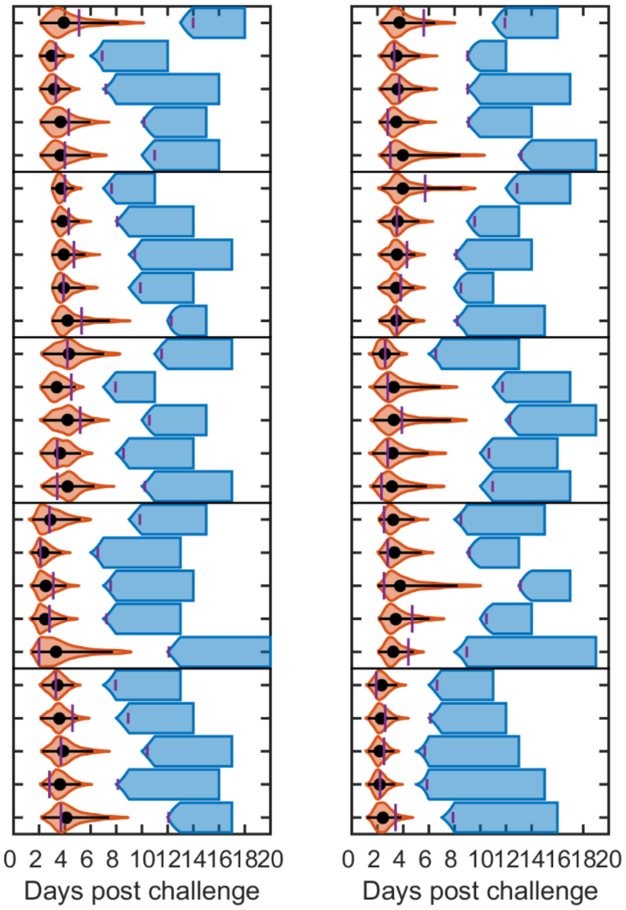

Figure S3. Inferred infection times for a synthetic 5 vs 5 transmission experiment. Orange violin plots show the posterior densities of the inferred infection times and the blue shapes represent the cumulative probability of each lamb being infectious at each time point. The black circles and bars denote the posterior median and the 95% highest posterior density interval. The longer purple lines mark the known infection times and the shorter purple lines indicate the start of the infectious period.

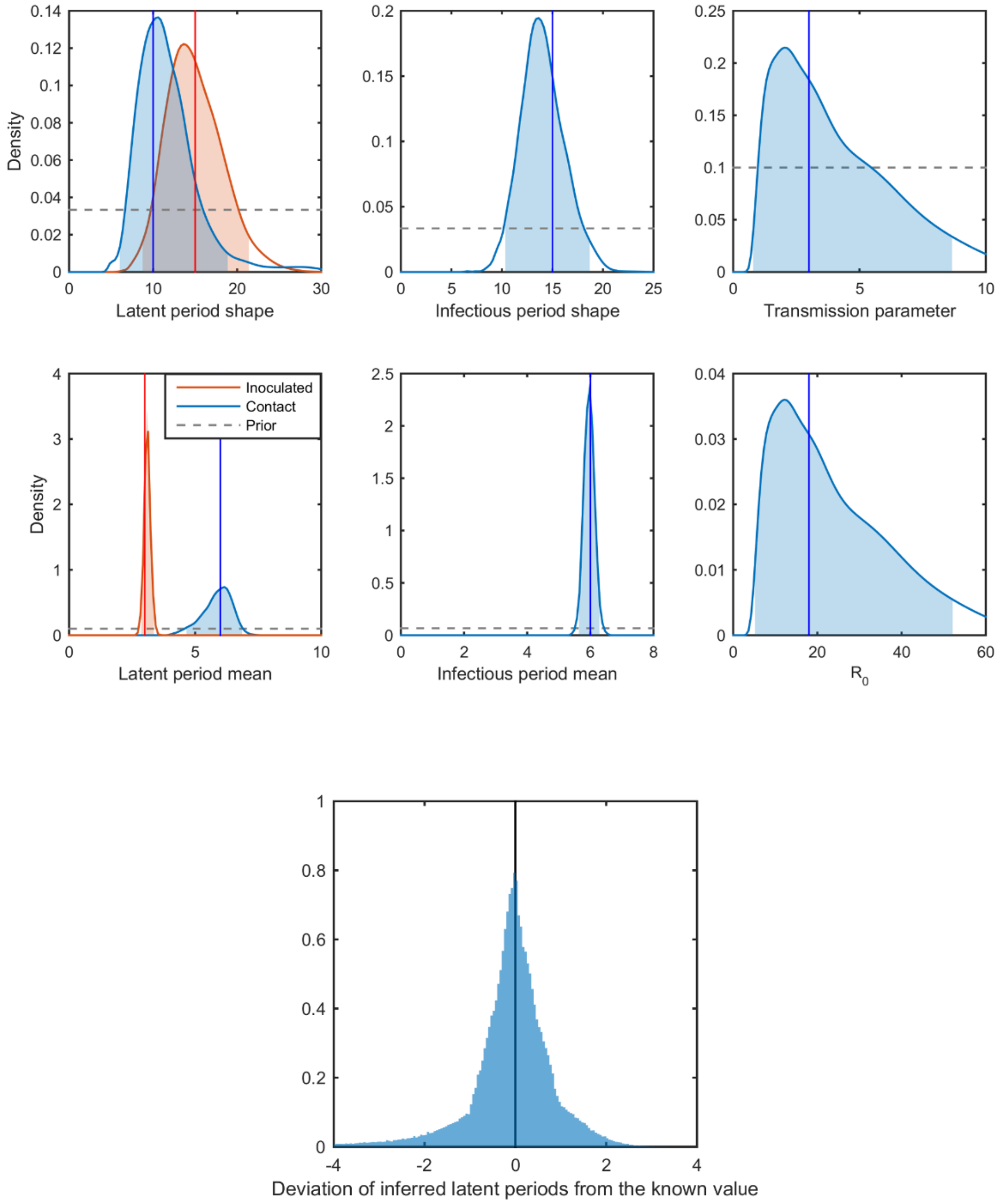

Figure S4. Inferred epidemiological parameters for a synthetic 5 vs 5 transmission experiment. Marginal posterior distributions for the parameters from the 5 vs. 5 synthetic dataset, with values used in the simulations indicated by vertical lines (top panels). The latent period shape and mean posteriors are plotted in blue for in-contact and red for inoculated animals. The shaded areas indicate the 95% highest posterior density intervals and priors are plotted as grey dashed lines. Histogram of deviations of the inferred infection times (bottom panel).

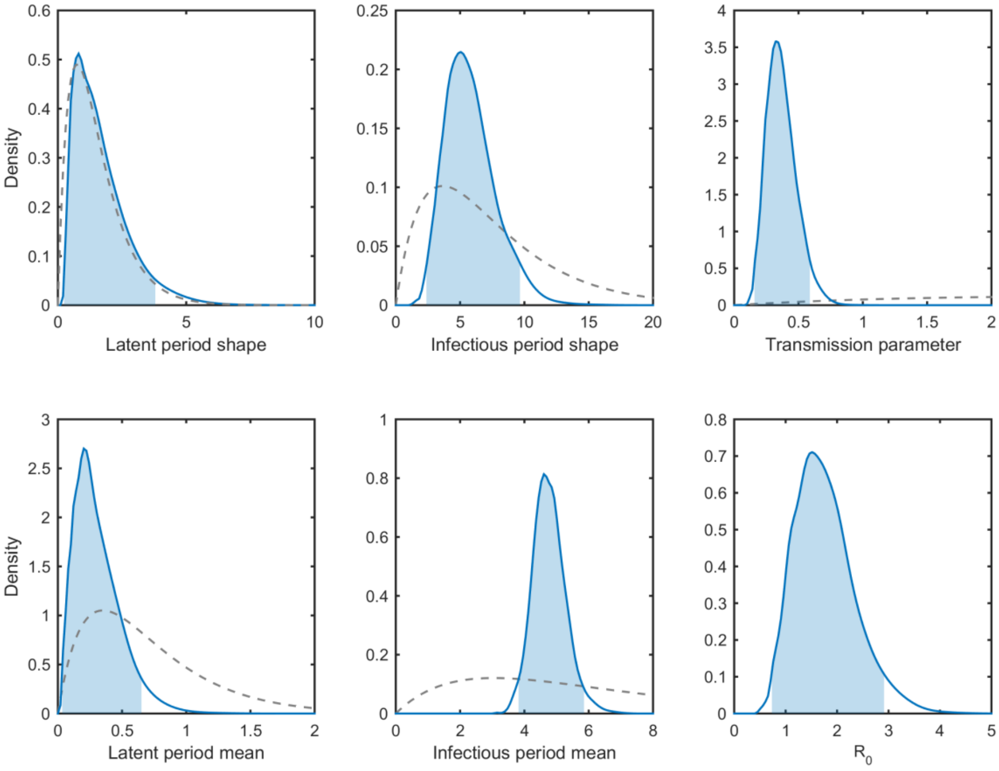

Figure S5. Epidemiological parameters for foot-and-mouth disease virus (FMDV) in vaccinated pigs. Marginal posterior distributions for each parameter inferred from the FMDV transmission experiments between vaccinated pigs. The posteriors are plotted as blue lines, the 95% highest posterior density intervals shown by the shaded area and the prior distributions are plotted as grey dashed lines throughout.

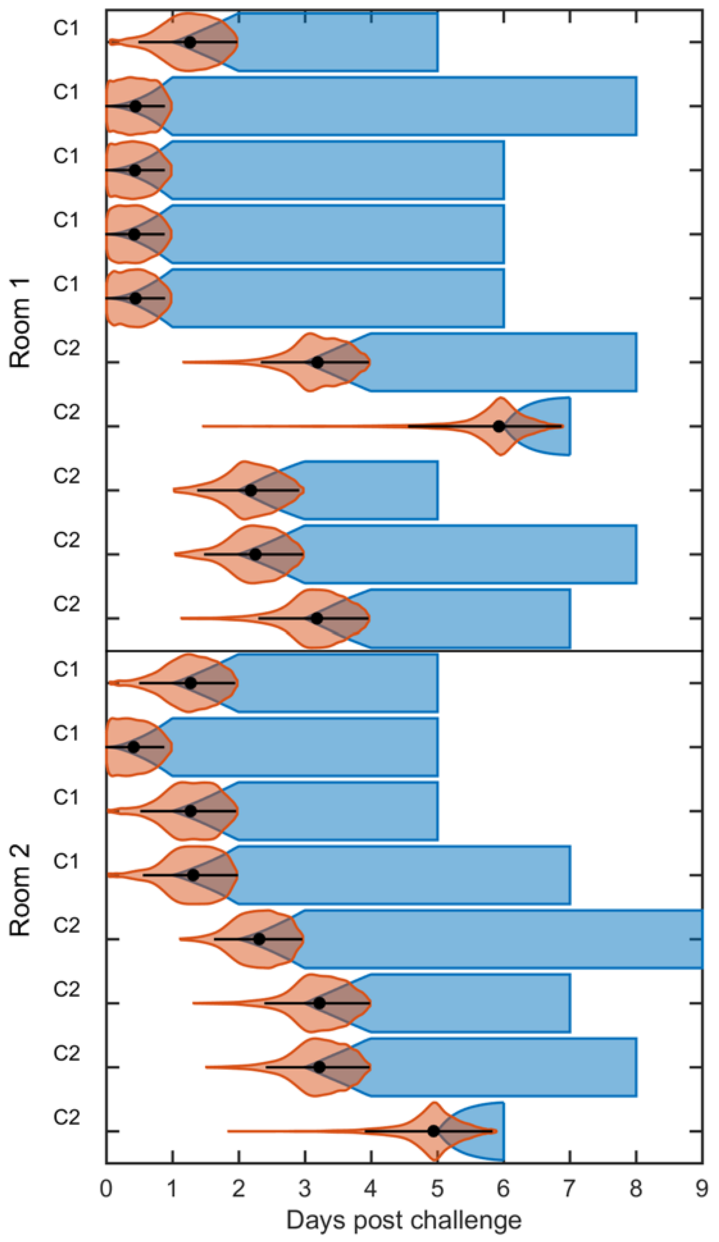

Figure S6. Inferred infection times for the FMDV in vaccinated pigs transmission experiment. Orange violin plots showing the densities of the inferred infection times for the unvaccinated contact pigs. The black circles and bars denote the posterior median and the 95% highest posterior density interval. The inferred cumulative probability of animals being infectious at each time point are shown in blue.

Appendix S1

Here we provide Matlab (version R2014b; The Mathworks, Inc.) code to implement the Bayesian methods. There are two functions `ImplementMCMCScheme.m` which loads the data and implements the adaptive Metropolis scheme and `Lhood.m` which computes the likelihood and prior for a given set of parameters.

The following implements the adaptive Metropolis scheme:

```
function ImplementMCMCScheme(lFlag, nchains,nsamp,nburnin,nthin)
%
% ImplementMCMCScheme(lFlag,nchains,nsamp,nburnin,nthin)
%
% Matlab function to find implement a Bayesian MCMC scheme to estimate
% transmission and latent, infectious and incubation period distribution
% parameters for FMDV based on transmission experiments.
%
% Inputs:
% lFlag - flag indicating whether latent periods are common (lFlag=1) or
%         differ (lFlag=2) between inoculated and contact animals
% nchains - number of chains to run
% nsamp - number of samples to use when estimating parameters
% nburnin - number of samples to discard before estimating parameters
% nthin - number of samples by which to thin each chain
%
% Outputs: none (N.B. All chains are saved rather than provided as output
% arguments)

% Initialise the random number generator
stream=RandStream('mt19937ar','Seed','Shuffle');
RandStream.setGlobalStream(stream);

%==========================================================================
% PREPARE THE DATA
% Load the experimental data
M=load('TransmissionExperimentData.txt');

% Extract the challange data. Vectors are:
% rList - room number (1-5)
% iType - infection type (1-inoculated, 2-contact)
% tLN - time of last negative result
% tFP - time of first positive result
% tLP - time of last positive result
% tFN - time of first negative result (after the last positive one)
rList=M(:,1);
iType=M(:,2);
tLN=M(:,3);
tFP=M(:,4);
tLP=M(:,5);
tFN=M(:,6);

% Compute the number animals with latent periods and infection times to
% infer
nLP=length(find(tLN>=0));
nI=length(find(iType>1 & tLN>=0));
%==========================================================================
```

```
% Set the number of parameters (i.e. model parameters and unobserved times
% of infection and latent periods for each animal)
if lFlag==1,
    nmp=5;
elseif lFlag==2,
    nmp=7;
end
npar=nI+nLP+nmp;

% Create the arrays storing the output for the chain
ParSamp=cell(1,nchains);
tISamp=cell(1,nchains);
LatPSamp=cell(1,nchains);

% For each chain ...
parfor chain=1:nchains,

%===========================================================================
% INITIALISE THE CHAIN
% Set the initial scaling factor for the proposal distribution
    sf=(2.38.^2)/npar;
    SIG=eye(npar);

% Set the counter for the number of accepted samples
    n_accept=0;

% Create the arrays storing the output for the chain
    ParSampC=zeros(nsamp/nthin,nmp+2);
    LatPSampC=zeros(nsamp/nthin,nLP+2);
    tISampC=zeros(nsamp/nthin,nI+2);
    iter=1;

% Generate the initial parameters for the chain, ensuring they generate a
% finite log likelihood and prior
    display('Initialising chain')
    CurrL=NaN;
    prior=NaN;
    while isfinite(CurrL)==0 || isfinite(prior)==0,

% Set the initial infection times and latent periods
        tI=unifrnd(tLN,tFP);
        tI(iType==1)=0;
        E=unifrnd(tI,tFP)-tI;
        tI=tI(tI>0);
        E=E(tLN>=0);

% Sample an initial set of parameters
        if lFlag==1,
            plat0=[unifrnd(0,5); unifrnd(0,2)];
        elseif lFlag==2,
            plat0=[unifrnd(0,5); unifrnd(0,2);
                   unifrnd(0,5); unifrnd(0,2)];
        end
        pinf0=[unifrnd(0,5); unifrnd(0,5)];
        b0=unifrnd(0,5);

% Combine the times of infection, latent periods and model parameters
        par=[tI; E; plat0; pinf0; b0];
```

```
% Compute the log-likelihood
        [CurrL, prior]=Lhood(par,tLN,tFP,tLP,tFN,iType,rList,lFlag);

    end
%===========================================================================

%===========================================================================
% ESTIMATE THE PARAMETERS
% Sample parameter space
    display('Sampling parameter space')
    for samp=1:nsamp+nburnin,

% Indicate what's going on
        display(['Chain: ' num2str(chain) ', Sample: ' num2str(samp) ';'...
                 ' Acceptance: ' num2str(100*n_accept/samp,3) '%'])

% Update the variance-covariance matrix for the proposal distribution
        if samp<=nburnin && (samp<=2*npar || n_accept==0),
            SIGp=0.001*eye(npar);
        else
            SIGp=sf.*(SIG+0.001*eye(npar));
        end

% Generate the new set of probabilities
        par_new=par+mvnrnd(zeros(1,length(par)),SIGp)';

% Compute the log likelihood and prior for the new parameter set
        [NewL, prior_new]=Lhood(par_new,tLN,tFP,tLP,tFN,iType,rList,lFlag);

% Test whether to accept the new parameter set
        u=unifrnd(0,1);
        if isfinite(NewL+prior_new)==1 && ...
           u<min(1,exp((NewL+prior_new)-(CurrL+prior))),

% Update the counter
            n_accept=n_accept+1;

% Update the covariance matrix for the proposal distribution
            if n_accept==1,
                pbar=mean([par par_new],2);
                SIG=cov([par'; par_new']);
            elseif samp<=nburnin && n_accept>1,
                pbar_prev=pbar;
                pbar=(n_accept./(n_accept+1)).*pbar_prev+...
                     (1./(n_accept+1)).*par_new;
                SIG=((n_accept-1)./n_accept).*SIG+...
                    (1./n_accept).*(n_accept.*(pbar_prev*pbar_prev')-...
                                    (n_accept+1).*(pbar*pbar')+...
                                    (par_new*par_new'));
            end

% Update the chain
            CurrL=NewL;
            prior=prior_new;
            par=par_new;

        end
```

```
% Every one hundred samples during burn-in, tune the scaling factor
% for the proposal distribution to ensure an acceptance rate of 20-40%
        if samp<=nburnin && mod(samp+1,100)==1 && n_accept/samp<0.2,
            sf=sf/2;
        elseif samp<=nburnin && mod(samp+1,100)==1 && n_accept/samp>0.4,
            sf=2*sf;
        end
%===========================================================================

%===========================================================================
% STORE THE OUTPUT
% After burn in, save iterations of the chain, thinning as specified
        if nthin==1,
            ParSampC(samp,:)=[par(nLP+nI+1:nLP+nI+nmp)' prior CurrL];
            LatPSampC(samp,:)=[par(nI+1:nI+nLP)' prior CurrL];
            tISampC(samp,:)=[par(1:nI)' prior CurrL];
        elseif samp>nburnin && mod(samp,nthin)==1,
            ParSampC(iter,:)=[par(nLP+nI+1:nLP+nI+nmp)' prior CurrL];
            LatPSampC(iter,:)=[par(nI+1:nI+nLP)' prior CurrL];
            tISampC(iter,:)=[par(1:nI)' prior CurrL];
            iter=iter+1;
        end
%===========================================================================

    end

% Store the chain
    ParSamp{chain}=ParSampC;
    LatPSamp{chain}=LatPSampC;
    tISamp{chain}=tISampC;

end

%===========================================================================
% COMPUTE THE DIC
% Compute the deviance for each sample
Dev=[];
PS=[];
for chain=1:nchains,
    Dev=[Dev; -2*ParSamp{chain}(:,end)];
    PS=[PS; [tISamp{chain}(:,1:end-2) ...
             LatPSamp{chain}(:,1:end-2) ...
             ParSamp{chain}(:,1:end-2)]];
end

% Compute the mean deviance
Dbar=mean(Dev);

% Compute the deviance at the posterior mean for the parameters
Dhat=-2*Lhood(mean(PS,1)',tLN,tFP,tLP,tFN,iType,rList,lFlag);

% Compute the DIC
DIC=2*Dbar-Dhat;

% Compute the effective number of parameters
pD=Dbar-Dhat;
%===========================================================================

%===========================================================================
```

```
% SAVE THE OUTPUTS
% Save to a file
save(['MCMCSamples_Model' num2str(lFlag)],...
     'ParSamp','LatPSamp','tISamp','nburnin','nsamp','DIC','pD')
%==========================================================================
```

The following computes the likelihood function for FMDV in lambs:

```
function [logL, prior]=Lhood(par,tLN,tFP,tLP,tFN,iType,rList,lFlag)
%
% [logL, prior]=Lhood(par,tLN,tFP,tLP,tFN,iType,rList,lFlag)
%
% Matlab function for computing the log likelihood and prior for
% transmission, latent and infectious period parameters for FMDV in lambs
% based on transmission experiments
%
% Inputs:
% par - array containing the model parameter
% tLN, tFP, tLP, tFN - vectors containing times of last negative,
%                      first positive, last positive result and first
%                      negative result, respectively
% iType - vector identifying infection type (1-inoculated, 2-contact)
% rList - vector containing room numbers
% lFlag - flag indicating whether latent periods are common (lFlag=1) or
%         differ (lFlag=2) between inoculated and contact animals
%
% Outputs:
% logL - log likelihood for parameters
% prior - log prior probability for parameters

%==========================================================================
% PREPARE THE INPUTS
% Compute the number of animals in the study
nLP=length(find(tLN>=0));
nI=length(find(iType>1 & tLN>=0));

% Set the infection times (all inoculated animals are assumed to be
% infected at time zero)
tI=NaN(size(iType));
tI(iType==1)=0;
tI(iType==2 & tLN>=0)=par(1:nI);

% Extract the unobserved latent periods
E=NaN(size(iType));
E(tLN>=0)=par(nI+1:nI+nLP);

% Extract the model parameters
if lFlag==1,
    p_lat=par(nI+nLP+1:nI+nLP+2);
    p_inf=par(nI+nLP+3:nI+nLP+4);
    b=par(nI+nLP+5);
elseif lFlag==2,
    p_latI=par(nI+nLP+1:nI+nLP+2);
    p_latC=par(nI+nLP+3:nI+nLP+4);
    p_inf=par(nI+nLP+5:nI+nLP+6);
    b=par(nI+nLP+7);
end
%==========================================================================
```

```
%========================================================================
% COMPUTE THE PRIOR
% Calculate the log(prior) for the parameters
if lFlag==1,
    prior=log(gampdf(p_lat(1),3,21/3))+...
          log(gampdf(p_lat(2),2,3/2))+...
          log(gampdf(p_inf(1),2,3/2))+...
          log(gampdf(p_inf(2),2,7.5/2))+...
          log(gampdf(b,2,2/2));
elseif lFlag==2,
    prior=log(gampdf(p_latI(1),3,21/3))+...
          log(gampdf(p_latI(2),2,3/2))+...
          log(gampdf(p_latC(1),3,21/3))+...
          log(gampdf(p_latC(2),2,3/2))+...
          log(gampdf(p_inf(1),2,3/2))+...
          log(gampdf(p_inf(2),2,7.5/2))+...
          log(gampdf(b,2,2/2));
end

% Constrain the latent periods to lie in appropriate ranges
prior=prior+...
      sum(log(unifpdf(tI(tLN>=0)+E(tLN>=0),tLN(tLN>=0),tFP(tLN>=0))));

% Constrain the infection times to lie in appropriate ranges
prior=prior+...
      sum(log(unifpdf(tI(iType==2 & tLN>=0),1,tFP(iType==2 & tLN>=0))));
%========================================================================

%========================================================================
% COMPUTE THE LOG LIKELIHOOD
% Initialise log-likelihood
logL=0;

% Set the timestep for computing the force of infection
dt=0.01;

% For each room ...
for r=1:max(rList),

% Extract the infection types, latent period, infection times and times of
% death for the experiment
    iTypeR=iType(rList==r);
    ER=E(rList==r);
    tIR=tI(rList==r);
    tLPR=tLP(rList==r);

% Set the time points for computing the probability of infection
    t=0:dt:ceil(max(tLPR));

% Compute the number of animals in the room
    n=length(iTypeR);

% Compute the number of infectious individuals at each time point
    I=zeros(size(t));
    for a=1:length(tIR),
        x=(tIR(a)+ER(a)<t & tLPR(a)>t);
        if isempty(x)==0,
            I(x)=I(x)+1;
```

```
        end
    end

% Compute the probability of infection for each animal
    for a=1:length(tIR),

% If the animal was not infected ...
        if isnan(tIR(a)),

% Calculate the probability of escaping infection ...
            p=exp(-(b.*sum(dt*I)/n));

% If the animal was infected (by contact, not inoculation) ...
        elseif tIR(a)>0,

% ... compute the probability of infection
            x=find(tIR(a)>=t,1,'last');
            if isempty(x)==0,
                p=(b.*I(x)./n).*exp(-(b.*sum(dt*I(1:x-1))/n));
            else
                p=0;
            end

        end

% Add the contribution of the probability of infection to the likelihood
        logL=logL+sum(log(p));

    end
end

% Add the contribution for the latent periods to the likelihood
if lFlag==1,
    logL=logL+sum(log(gampdf(E(~isnan(E)),p_lat(1),p_lat(2)/p_lat(1))));
elseif lFlag==2,
    logL=logL+sum(log(gampdf(E(~isnan(E) & iType==1),...
                             p_latI(1),p_latI(2)/p_latI(1))))+...
              sum(log(gampdf(E(~isnan(E) & iType==2),...
                             p_latC(1),p_latC(2)/p_latC(1))));
end

% Add the contribution for the infectious periods to the likelihood
x=(~isnan(E) & ~isnan(tFN));
logL=logL+sum(log(gamcdf(tFN(x)-tI(x)-E(x),p_inf(1),p_inf(2)/p_inf(1))-...
                  gamcdf(tLP(x)-tI(x)-E(x),p_inf(1),p_inf(2)/p_inf(1))));
x=(~isnan(E) & isnan(tFN));
logL=logL+sum(log(1-gamcdf(tLP(x)-tI(x)-E(x),p_inf(1),p_inf(2)/p_inf(1))));
%===========================================================================
```

The following computes the likelihood for the ASFV in pigs experiment.

```
function [logL, prior]=Lhood(par,tLN,tFP,tD,iType,rList,lFlag)
%
% [logL, prior]=Lhood(par,tLN,tFP,tD,iType,rList,lFlag)
%
% Matlab function for computing the log likelihood and prior for
% transmission, latent and infectious period parameters for ASFV based on
```

```
% transmission experiments.
%
% Inputs:
% par - array containing the (transformed) model parameter
% tLN, tFP, tD - vectors containing times of last negative result,
%                times of first positive result and times of death,
%                respectively
% iType - vector identifying infection type (1-inoculation, 2-within-pen
%         contact, 3-between-pen contact)
% rList - vector containing room numbers (1-4)
% lFlag - flag indicating whether latent periods are common (lFlag=1) or
%         differ (lFlag=2) between inoculated and contact animals
%
% Outputs:
% logL - log likelihood for parameters
% prior - log prior probability for parameters

%==========================================================================
% PREPARE THE INPUTS
% Compute the number of animals in the study and the number of contacts
nA=length(iType);
nC=length(find(iType>1));

% Set the infection times (all inoculated animals are assumed to be
% infected at time zero)
tI=zeros(nA,1);
tI(iType>1)=par(1:nC);

% Extract the unobserved latent periods
E=par(nC+1:nC+nA);

% Extract the model parameters
if lFlag==1,
    p_lat=par(nC+nA+1:nC+nA+2);
    p_inf=par(nC+nA+3:nC+nA+4);
    bW=par(nC+nA+5);
    bB=par(nC+nA+6);
elseif lFlag==2,
    p_latI=par(nC+nA+1:nC+nA+2);
    p_latC=par(nC+nA+3:nC+nA+4);
    p_inf=par(nC+nA+5:nC+nA+6);
    bW=par(nC+nA+7);
    bB=par(nC+nA+8);
end
%==========================================================================

%==========================================================================
% COMPUTE THE PRIOR
% Calculate the log(prior) for the parameters
if lFlag==1,
    prior=log(gampdf(p_lat(1),2,10/2))+...
          log(gampdf(p_lat(2),10,4.5/10))+...
          log(gampdf(p_inf(1),2,19.33/2))+...
          log(gampdf(p_inf(2),10,5.96/10))+...
          log(gampdf(bW,2,1))+...
          log(gampdf(bB,2,1));
elseif lFlag==2,
    prior=log(gampdf(p_latI(1),2,10/2))+...
          log(gampdf(p_latI(2),10,4.5/10))+...
          log(gampdf(p_latC(1),2,10/2))+...
```

```
          log(gampdf(p_latC(2),10,4.5/10))+...
          log(gampdf(p_inf(1),2,19.33/2))+...
          log(gampdf(p_inf(2),10,5.96/10))+...
          log(gampdf(bW,2,1))+...
          log(gampdf(bB,2,1));
end

% Constrain the latent periods to lie in appropriate ranges
prior=prior+sum(log(unifpdf(tI+E,tLN,tFP)));

% Constrain the infection times to lie in appropriate ranges
prior=prior+sum(log(unifpdf(tI,0,tFP)));
%==========================================================================

%==========================================================================
% COMPUTE THE LOG LIKELIHOOD
% Initialise log-likelihood
logL=0;

% Set the timestep for computing the force of infection
dt=0.01;

% For each room ...
for r=1:max(rList),

% Extract the infection types, latent period, infection times and times of
% death for the experiment
    iTypeR=iType(rList==r);
    ER=E(rList==r);
    tIR=tI(rList==r);
    tDR=tD(rList==r);

% Set the time points for computing the probability of infection
    t=0:dt:ceil(max(tIR));

% Compute the number of infectious individuals and total number of
% individual within each pen at each time point
    IW=zeros(size(t));
    nW=zeros(size(t));
    for a=1:length(tIR),
        x=(tIR(a)+ER(a)<t & tDR(a)>t & iTypeR(a)<=2);
        if isempty(x)==0,
            IW(x)=IW(x)+1;
        end
        x=(tDR(a)>t & iTypeR(a)<=2);
        if isempty(x)==0,
            nW(x)=nW(x)+1;
        end
    end
    IB=zeros(size(t));
    nB=zeros(size(t));
    for a=1:length(tIR),
        x=(tIR(a)+ER(a)<t & tDR(a)>t & iTypeR(a)==3);
        if isempty(x)==0,
            IB(x)=IB(x)+1;
        end
        x=(tDR(a)>t & iTypeR(a)==3);
        if isempty(x)==0,
            nB(x)=nB(x)+1;
        end
```

```
   end

% Compute the probability of infection for each pig
    pI=ones(size(tIR));
    for a=1:length(pI),

% If the pig is a within-pen contact ...
        if iTypeR(a)==2,

% Determine the time of infection
            x=find(tIR(a)>=t,1,'last');

% Compute the probability of infection at that time
            if isempty(x)==0,
                pI(a)=(bW.*IW(x)./nW(x)+bB.*IB(x)./(nW(x)+nB(x))).*...
                       exp(-(bW.*sum(dt*IW(1:x-1)./nW(1:x-1))+...
                             bB.*sum(dt*IB(1:x-1)./...
                                        (nW(1:x-1)+nB(1:x-1)))));
            else
                pI(a)=0;
            end

% If the pig is a between-pen contact ...
        elseif iTypeR(a)==3,

% Determine the time of infection
            x=find(tIR(a)>=t,1,'last');

% Compute the probability of infection at that time
            if isempty(x)==0,
                pI(a)=(bW.*IB(x)./nB(x)+bB.*IW(x)./(nW(x)+nB(x))).*...
                       exp(-(bW.*sum(dt*IB(1:x-1)./nB(1:x-1))+...
                             bB.*sum(dt*IW(1:x-1)./...
                                        (nW(1:x-1)+nB(1:x-1)))));
            else
                pI(a)=0;
            end

        end
    end

% Add the contribution of the probability of infection to the likelihood
    logL=logL+sum(log(pI));

end

% Add the contribution for the latent periods to the likelihood
if lFlag==1,
    logL=logL+sum(log(gampdf(E,p_lat(1),p_lat(2)/p_lat(1))));
elseif lFlag==2,

logL=logL+sum(log(gampdf(E(iType==1),p_latI(1),p_latI(2)/p_latI(1))))...
             +sum(log(gampdf(E(iType>1),p_latC(1),p_latC(2)/p_latC(1))));
end

% Add the contribution for the infectious periods to the likelihood
logL=logL+sum(log(1-gamcdf(tD-E-tI,p_inf(1),p_inf(2)/p_inf(1))));
%===========================================================================
```

Data S1

Below are the data for the foot-and-mouth disease virus (FMDV) in lambs experiments. Columns are:

1. room number
2. infection type (coded as 1 for inoculated and 2 for contact-challenged)
3. day of last negative sample (set to -1 if always negative)
4. day of first positive sample (set to -1 if always negative)
5. day of last positive sample
6. time of first negative sample (set to `NaN` if no first negative sample)

Note, the inoculated lambs are challenged on day zero.

1	2	-1	-1	14	NaN
1	2	-1	-1	14	NaN
1	1	1	2	12	13
1	1	-1	-1	14	NaN
2	2	-1	-1	14	NaN
2	2	-1	-1	14	NaN
2	1	0	1	14	NaN
2	1	2	3	14	NaN
3	2	-1	-1	14	NaN
3	2	-1	-1	14	NaN
3	1	0	1	14	NaN
3	1	1	2	12	13
4	2	7	8	14	NaN
4	2	4	5	14	NaN
4	1	0	1	13	14
4	1	0	1	12	13
5	2	-1	-1	14	NaN
5	2	-1	-1	14	NaN
5	1	0	1	14	NaN
5	1	2	3	10	11
6	2	-1	-1	14	NaN
6	2	3	4	13	14
6	1	0	1	14	NaN
6	1	0	1	14	NaN

Data S2

Below are the data for the foot-and-mouth disease virus (FMDV) in pigs experiments. Columns are:

1. room number
2. infection type (coded as 1 for inoculated, 2 for contact-challenged (C1) and 3 for contact-challenged (C2))
3. day of last negative sample
4. day of first positive sample
5. day of last positive sample
6. censoring for the last positive sample (coded as 0 if no and 1 if yes)

Note: the inoculated pigs are challenged on day zero. In addition, the time of first negative sample is always one day after the last positive sample, unless censored.

1	2	2	3	7	0
1	2	1	2	5	1
1	2	1	2	6	0
1	2	1	2	4	0
1	2	1	2	6	0
1	3	2	3	9	0
1	3	3	4	11	0
1	3	2	3	7	0
1	3	2	3	8	0
1	3	3	4	8	1
2	2	1	2	6	0
2	2	1	2	5	1
2	2	1	2	5	1
2	2	2	3	6	0
2	2	3	4	7	0
2	3	2	3	9	0
2	3	2	3	8	0
2	3	2	3	8	0
2	3	3	4	9	0
2	3	2	3	6	0
3	1	2	3	9	0
3	2	2	3	9	0
4	1	0	1	4	0
4	2	1	2	6	0
5	1	0	1	8	0
5	2	1	2	6	0
6	1	0	1	5	0
6	2	1	2	7	0
7	1	1	2	9	0
7	2	1	2	7	0

Data S3

Below are the data for the African swine fever virus (ASFV) in pigs experiments. Columns are:

1. room number
2. infection type (coded as 1 for inoculated, 2 for within-pen contact and 3 for between-pen contact)
3. day of last negative sample
4. day of first positive sample
5. day of death

Note: the inoculated pigs are challenged on day zero.

1	1	3	5	9
1	1	0	3	9
1	1	0	3	7
1	1	0	3	6
1	1	3	5	8
1	2	9	11	12
1	2	11	13	13
1	2	9	11	13
1	2	9	11	13
1	2	9	11	13
2	1	0	3	6
2	1	3	5	8
2	1	0	3	6
2	1	3	5	6
2	2	7	9	10
2	2	7	9	10
2	2	7	9	10
2	2	7	9	10
2	3	9	11	13
2	3	11	13	15
2	3	9	11	15
2	3	7	9	11
3	1	0	3	8
3	1	0	3	7
3	1	3	5	12
3	1	0	3	6
3	2	7	9	12
3	2	9	11	12
3	2	7	9	10
3	2	11	13	14
3	3	17	18	18
3	3	15	17	18
3	3	11	13	16
3	3	11	13	15
4	1	0	3	6
4	1	0	3	7
4	1	0	3	6
4	2	7	9	10
4	2	9	11	13
4	2	9	11	13
